# Applying Chitosan-Based Films Enriched with *Borago officinalis* Extract for Active and Green Packaging of Fresh Rainbow Trout Fillets

**DOI:** 10.3390/foods14040639

**Published:** 2025-02-14

**Authors:** Alper Güngören, Yasin Akkemik, Enis Fuat Tufekci, Gökhan Zengin, Gizem Emre, Gulsah Gungoren, Yasemin Celik Altunoglu, Mehmet Cengiz Baloğlu

**Affiliations:** 1 Department of Food Hygiene and Technology, Faculty of Veterinary Medicine, Kastamonu University, 37150 Kastamonu, Türkiye; yakkemik@kastamonu.edu.tr; 2 Department of Medical Microbiology, Faculty of Medicine, Kastamonu University, 37150 Kastamonu, Türkiye; etufekci@kastamonu.edu.tr; 3 Department of Biology, Science Faculty, Selcuk University, 42130 Konya, Türkiye; gokhanzengin@selcuk.edu.tr; 4 Department of Pharmaceutical Biology, Pharmacy Faculty, Marmara University, 34722 Istanbul, Türkiye; gizem.bulut@marmara.edu.tr; 5 Department of Animal Science, Faculty of Veterinary Medicine, Kastamonu University, 37150 Kastamonu, Türkiye; ggungoren@kastamonu.edu.tr; 6 Plantomics Research Laboratory, Department of Genetics and Bioengineering, Faculty of Engineering and Architecture, Kastamonu University, 37150 Kastamonu, Türkiye; ycaltunoglu@kastamonu.edu.tr (Y.C.A.); mcbaloglu@kastamonu.edu.tr (M.C.B.)

**Keywords:** *Oncorhynchus mykiss*, borage, edible film, ethanolic extract, shelf life, reduce food waste

## Abstract

This study aimed to apply chitosan (CS) coating films enriched with *Borago officinalis* extract to preserve fresh rainbow trout fillets. Extracts of *B. officinalis* were prepared using ethyl acetate, ethanol, water, and an ethanol-water mixture. These extracts were incorporated into chitosan coating films at 0.5% and 1% (*v*/*v*) concentrations, and their antimicrobial activity and antioxidant abilities were investigated. CS films with borage extract in ethanol-water combination showed the highest antibacterial zone diameter (9.5 ± 2.1 mm) against *Staphylococcus aureus*. Based on its superior antimicrobial and antioxidant activity, the ethanol-water extract was selected for further film characterization, including moisture content, swelling degree, solubility, and color. The films were then tested for their effectiveness in preserving rainbow trout fillets stored at 7 ± 1 °C. The fish samples were divided into four groups: control, chitosan coating film, chitosan coating film with 0.5% *B. officinalis* extract, and chitosan coating film with 1% *B. officinalis* extract. Physicochemical, chemical, and microbiological analyses were conducted until fillet spoilage was observed (12 days). Results demonstrated that chitosan coating films enriched with 1% extract of *B. officinalis* prolonged the expiration date of the fish by six days, had significant antioxidant properties, and protected fish from discoloration. While the coating films demonstrated promising antioxidant properties and the shelf life extension of the fish samples by six days, further optimization will be required to enhance their antimicrobial effectiveness.

## 1. Introduction

Fish meat and fishery products are recognized as highly nutritious and healthy foods. Yet, it is widely known that seafood is particularly prone to bacterial spoilage, lipid oxidation, and autolytic and enzymatic spoilage due to its high water activity, relatively neutral pH, high unsaturated fat content, and microbiota [1,2]. Appropriate technology and infrastructure are essential to prevent this rapid degradation and loss of quality. Packaging is critical to the fish supply chain and key to addressing food waste [3]. The slow decomposition of petroleum-based plastic packaging films by nature is a significant obstacle to achieving sustainable global development plans. Biodegradable chitosan polymers have been developed as a sustainable option for non-biodegradable polymeric materials in various packaging applications [4,5].

Fish meat cannot be stored at refrigerator temperature (4 °C) for a long time. In addition, in many homes, refrigerators do not work efficiently and their doors are opened and closed frequently, so the refrigerator temperature can rise to 6–8 °C or even higher. There is a limited time for fish to be consumed after purchase. Because of this, one of the present issues in fish meat preservation is the development of active and green packaging materials. Active packaging is the materials and components intended to maintain or improve the packaged food’s conditions or extend its shelf life. Active packaging could incorporate antimicrobial and antioxidant compounds inside the packaging materials [6,7,8]. Chitosan is a popular structure-building material for carbohydrate-based edible packaging films. It is mainly obtained through alkaline N-deacetylation of chitin, which is derived from waste from the shellfish (shrimp, crabs, lobsters, etc.) industry. Its linear polysaccharide structure has nontoxic properties, biodegradability, and functionality [8,9].

The growing consumer trend is increasing the demand for high-quality foods that are environmentally sustainable, free from synthetic chemical preservatives, and have an extended shelf life [9,10]. Various herbal compounds became popular due to consumers’ demand for natural food additives that are healthier and preferable to chemical-based ones [3,9]. Borage (*Borago officinalis* L., *Boraginaceae*) or starflower, is a Mediterranean-native annual herb utilized for culinary purposes since ancient times. Vegetable use of borage is common in Germany, Italy, Spain, Türkiye, and Iran. Moreover, multiple studies have demonstrated the antimicrobial, antioxidant potential, and significant phenolic content of *B. officinalis* [11,12,13,14].

Previously, chitosan-based films combined with various compounds have been used to prolong the shelf life and elevate the quality of fresh aquatic products [9,15,16]. The aforementioned initiatives also agree with consumers’ demand for minimally processed or organic food products. Nevertheless, to our knowledge, the literature addressing the implications of chitosan-based films with *B. officinalis* extract is limited. Thus, this research focused on applying chitosan-based coating films enriched with *B. officinalis* extracts for active and green packaging of fresh Rainbow trout fillets. Additionally, current research is aimed at investigating the impact of this film on the shelf life of rainbow trout fillets in refrigerator circumstances.

## 2. Material and Methods

### 2.1. Plant Biomaterials and Extractions

In 2022, botanical specimens were collected from the Maltepe, Başıbüyük area in Istanbul, Türkiye. Dr. Gizem Emre conducted the taxonomic identification and a voucher specimen was preserved in the herbarium of the Pharmacy Faculty at Marmara University (voucher number: MARE-22677). The aerial parts were separated, dried in the shade at ambient temperature, pulverized, and stored away from light. The extraction procedure included four solvents: ethyl acetate, ethanol, a 70% ethanol/water mixture, and water. Each 10 g sample was macerated with 200 mL of ethyl acetate, ethanol, and a mixture of ethanol and water for 24 h at ambient temperature. The aqueous extract was obtained by infusing 10 g of the *B. officinalis* plant in boiling water for fifteen minutes. Organic solvents were taken out via evaporation under low pressure, and the aqueous extract was subjected to freeze-drying. The collected extracts were stored at room temperature in the dark for chitosan film production.

### 2.2. Production of Films

The chitosan-based film was prepared by mixing chitosan (CS) (2% *w*/*v*, >75% deacetylated, high molecular weight, Sigma Chemical Co., St. Louis, MO, USA) in glacial acetic acid aqueous solution (2.0%, *v*/*v*) with magnetic stirring at 60 °C for 60 min. Subsequently, glycerol (40%, *w*/*w*) was included, performing as a plasticizing agent, followed by continued stirring at 60 °C for 30 min. After that, different concentrations of all extracts (0.5% and 1%, hereafter referred to as CS + 05 and CS + 1, respectively) were added into the chitosan film-forming solutions with continuous stirring at 60 °C for 30 min. The control CS film was made without the borage extract. Thus, the solutions were prepared with different concentrations of extracts obtained from ethyl acetate, ethanol, water, and ethanol-water mixtures as solvents were poured 15 mL into 90 mm diameter petri dishes. The resulting nine group solutions were left to dry at 25 °C and 50–60% relative humidity for 48 h. The films obtained after drying were stored in a desiccator for further analysis.

#### 2.2.1. Antioxidant Capacity of Films

The absorbance of the film samples (20 mg) and 4 mL of DPPH-ethanol solution was measured at 517 nm absorbance using a UV-1800 spectrophotometer (Shimadzu, Tokyo, Japan) and the radical scavenging activity was determined using Equation (1).

(1) $$DPPH\;\;Scavenging\;\;activity\;\left(\%\right)=\left[\frac{A1-A2}{A1}\times 100\right]$$

in which *A*1 represents the absorbance of the 95% ethanol with DPPH and *A*2 represents the absorbance of the samples with DPPH [17].

#### 2.2.2. Antimicrobial Activity of Films

The antimicrobial activity of the films was surveyed against three Gram-positive bacteria (*Bacillus subtilis* ATCC 6633, *Enterococcus faecalis* ATCC 29212, and *Staphylococcus aureus* ATCC 25923), five Gram-negative bacteria (*Acinetobacter haemolyticus* ATCC 19002, *Escherichia coli* ATCC 25922, *Klebsiella pneumoniae* ATCC 13883, *Pseudomonas aeruginosa* ATCC 27853, and *Salmonella* Typhimurium ATCC 14028), and one yeast (*Candida albicans* ATCC 10231) strains representing pathogens. In brief, inocula with a density of 0.5 McFarland were prepared from fresh microbial cultures. The inocula were spread onto Mueller-Hinton agar (Biolife, Milan, Italy) plates in three directions using sterile cotton swabs. CS films incorporated with plant extracts, with an approximate diameter of 6 mm, were then placed onto the agar surface. Levofloxacin (5 µg/disc) and nystatin (100 U/disc) were used as positive controls for bacteria and yeast, respectively, while CS film without any additives served as negative control. Following incubation at 37 ± 1 °C for 24 h, the inhibition zone diameters around the films were measured using a millimeter ruler. The process was carried out following EUCAST guidelines [18].

### 2.3. Characterization of Selected Films

According to the antimicrobial and antioxidant results, the most reasonable films were selected, and their physical properties were determined.

#### 2.3.1. Determination of Moisture Content, Swelling Degree, and the Solubility of Films

These analyses were performed using gravimetric methods. The total moisture content of the films was determined by the oven drying method at 105 ± 1 °C until it reached a stable weight. Then, moisture was determined using Equation (2).

(2) $$Moisture\left(\%\right)=\frac{M1-M2}{M1}\times 100$$

in which *M*1 represents the initial weight, and *M*2 represents the dry weight of films. For film solubility analyses, film pieces were sliced into 2 × 2 cm sizes, and solubility was determined using Equation (3).

(3) $$Solubility\left(\%\right)=\frac{S1-S2}{S1}\times 100$$

in which *S*1 represents the initial dry weight and *S*2 represents the final dry weight after placing the films in 50 mL distilled water for 24 h and drying at 105 ± 1 °C. Also, Equation (4) was used to calculate the swelling degree.

(4) $$Swelling\;\;degree\left(\%\right)=\frac{Sd2-Sd1}{Sd1}\times 100$$

in which *Sd*1 represents the initial weight. *Sd*2 represents the final weight after placing the films in 30 mL distilled water for 24 h [19].

#### 2.3.2. Film Colors

A colorimeter device was used to analyze the color properties of film samples (CS-10, CHNSpec, Hangzhou, China). The results were presented in color parameters L*, which is lightness/darkness; a*, which is redness/greenness; and b*, as well as yellowness/blueness. In each sample, at least four spots were measured for color at room temperature. Then, Equations (5)–(10) were used to determine the total color difference (ΔE), whiteness index (WI), browning index (BI), yellowness index (YI), chroma (C*), and hue angle (h*), respectively.

(5) $$Total\;\;color\;\;difference\;(\Delta E)=\sqrt{{\left(\Delta L*\right)}^{2}{+(\Delta a*)}^{2}+({\Delta b*)}^{2}}$$

(6) $$Whiteness\;\;index\;(WI)=100-\sqrt{{(100-L*)}^{2}{+(a*}^{2}+{b*}^{2})}$$

(7) $$Browning\;\;index\;(BI)=\frac{100\times \left(\left(\frac{a*\;+\;1.75\;\times \;L*}{5.645\;\times \;L*\;+\;a*\;-\;0.3012\;\times \;b*}\right)-0.31\right)}{0.17}$$

(8) $$Yellowness\;\;index\;(YI)=\frac{142.86\times b*}{L*}$$

(9) $$Chroma\;(C*)=\sqrt{{(a*)}^{2}+{(b*)}^{2}}$$

(10) $$Hue\;\;angle\;(h*)={tan}^{-1}\left(\frac{b*}{a*}\right)$$

### 2.4. Coating Film Application of Rainbow Trout Fillets

A local fish farmer provided rainbow trout (*Oncorhynchus mykiss*, Walbaum 1792, https://www.marinespecies.org/aphia.php?p=taxdetails&id=127185, accessed on 15 January 2025), each weighing 250 ± 50 g (Kastamonu, Türkiye). Immediately after being placed in a cold storage box, the fresh fish was taken to the laboratory. Subsequently, the fresh fish were decapitated, eviscerated, washed, and filleted. A total of 22 fish were used in each replication for microbiological and chemical examination, resulting in 66 fish (132 fillets) after three repetitions. All fillet samples were separated into four groups (C: without treatment, CS: immersed in chitosan solution, CS + 05: immersed in chitosan solution with 0.5% (*v*/*v*) *B. officinalis* extract, CS + 1: immersed in chitosan solution with 1% (*v*/*v*) *B. officinalis* extract). Each rainbow trout was dipped in a chitosan treatment solution for 5 min and allowed to air dry for 30 min in a fan cooler at 10 ± 1 °C. Then, each fillet was placed into a foam plate and in a nylon bag. The control group was packaged in the same way without any treatment. After that, all products were stored at 7 ± 1 °C before analysis on days 0, 3rd, 6th, 9th, and 12th.

### 2.5. Physicochemical Characteristics of Fish Samples

The moisture content, crude ash, crude fat, and protein content of the samples were quantified using procedures following AOAC specifications [20]. The pH meter (HI, 11310, Hanna Instruments, Woonsocket, RI, USA) was used to measure the pH of the samples. pH measurements were performed on the stomacher bags following microbiological analyses. Color analysis was also performed on fish samples, as stated above for film color. To determine cooking loss (CL), the fish muscle was taken from the same location of each fish for the analyses. The fish meat sample was weighed (*C*1) and placed into a sampling bag. It was then cooked in a water bath maintained at 85 ± 2 °C for 10 min. After ten min, the excess moisture from the samples was removed with hydrophilic gauze and the final weight (*C*2) was determined [21]. CL was determined using Equation (11).

(11) $$Cooking\;\;loss\;(CL)=\frac{C2-C1}{C2}\times 100$$

Centrifugal loss is designated as water-holding capacity (WHC). Briefly, a sample of about 2 g (*W*1) was placed in a falcon tube that contained cotton in the bottom. Tubes were centrifuged at 1.590× *g* for 10 min. After centrifugation. The sample was weighted (*W*2). Then, the WHC was determined using Equation (12).

(12) $$Water\;\;holding\;\;capacity\;(WHC)=100-\left(\frac{W1-W2}{W1}\times 100\right)$$

### 2.6. Chemical Analyses

#### 2.6.1. Determination of Total Volatile Base-Nitrogen (TVB-N)

A quantity of ten grams of the sample was added to 100 mL of distilled water, followed by homogenization using a stomacher mixer. This homogenate was poured into the Kjeldahl tube. Two grams of magnesium oxide and several drops of antifoaming compounds were added to the tubes. Following this, 200 mL of distillate was collected in an Erlenmeyer flask holding 25 mL of a 3% boric acid solution and a mL of Tashiro’s indicator. After the distillation, titration was carried out using 0.1 mol equi/L HCl solution until the neutralization occurred. Results of the TVB-N quantity in fillet samples were calculated by using Equation (13) and are expressed in mg per 100 g.

(13) $$Total\;\;volatile\;\;base-nitrogen\;(TVB-N)=\frac{1.4\times V}{G}\times 100$$

in which *V* represents 0.1 mol equi/L HCl volume. *G* represents the sample weight [22].

#### 2.6.2. Determination of Free Fatty Acids (FFA) Analyses

A sample weighing ten grams was homogenized with 30 mL of chloroform containing 0.5 g of Na_2_SO_4_ and let to settle at 20 °C for five minutes. The solution was then filtered. Thereafter, 25 mL of the filtrate was mixed with 25 mL of ethanol. The free fatty acids in 50 mL of the solution were titrated using a 0.1 mol equi/L NaOH solution [22,23]. Results of the FFA in samples were calculated by using Equation (14) and presented as mg/100 g.

(14) $$Free\;\;fatty\;\;acids\;(FFA)=\frac{V\times 2.82}{T}$$

where *V* is the volume of titration. *T* is the weight of the sample.

#### 2.6.3. Thiobarbituric Acid (TBA) Analyses

A sample of ten grams of homogenized fillet was mixed with 50 mL of distilled water and transferred into a Kjeldahl tube. Subsequently, an additional 47.5 mL of distilled water and 2.5 mL of hydrochloric acid solution were introduced to adjust the pH to 1.5. Distillation was then conducted until a total of 50 mL of distillate was obtained. Following this, 5 mL of the distillate was blended with 5 mL of thiobarbituric acid (TBA) reagent and heated for 35 min. The absorbance was measured at a wavelength of 532 nm, using a blank reagent for comparison. Lastly, the recorded absorbance measurements were multiplied by a factor (7.8) to convert to milligrams of malonaldehyde (MDA) per kilogram [24].

### 2.7. Microbiological Analyses

On each analysis day, 10 g from each sample package was collected and transferred meticulously to a sterile sampling bag containing 90 mL of 0.1% sterilized peptone water. The mixtures were homogenized for 120 s using a stomacher to ensure a uniform suspension. For the assessment of aerobic plate counts (APC), Plate Count Agar (PCA) was utilized, with incubation set at 35 ± 1 °C for 24 to 48 h. Psychrotrophic bacteria (PB) were evaluated by maintaining samples at a temperature of 5–7 °C for a duration of 7 to 10 days. To determine the presence of mesophilic lactic acid bacteria (LAB), de Man Rogosa Sharpe Agar (MRS) was employed, with incubation conditions of 30 ± 1 °C for 72 h. Furthermore, yeast and mold enumeration was conducted using Dichloran Rose Bengal Chloramphenicol (DRBC) Agar, with samples incubated at 25 ± 1 °C for 5 days. The results obtained are reported as log_10_ CFU/g.

### 2.8. Statistical Analyses

In the study, all data were collected from three independent replicates and presented as mean ± standard deviation (m ± sd). To assess the significance between groups, the Tukey multiple comparison test (*p* < 0.05) was employed following variance (ANOVA) analysis. Statistical analyses were conducted using the statistical computer program named SPSS 21.0.

## 3. Results and Discussion

### 3.1. In Vitro Antioxidant and Antimicrobial Activity of CS Films

**Figure 1:**
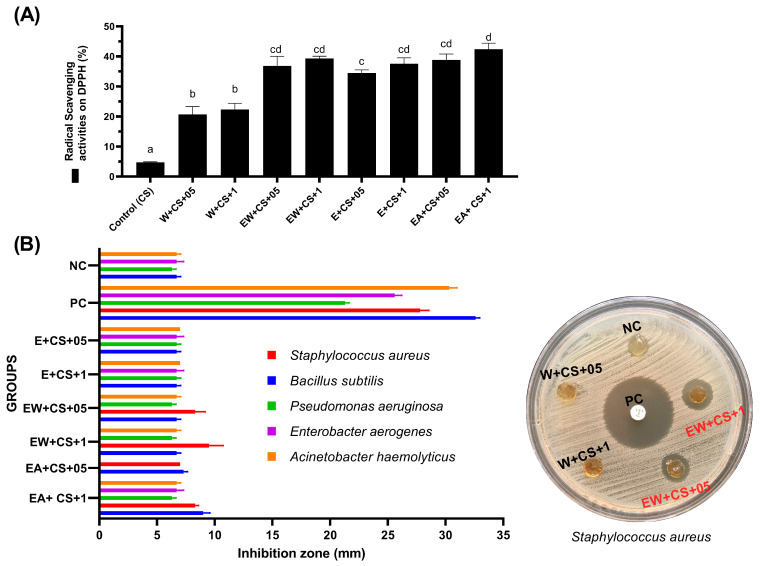
(**A**) The antioxidant activity of the chitosan (CS) films formulated using ethyl acetate (EA), ethanol (E), water (W), and a combination of ethanol-water (EW) of *B. officinalis* extracts, each of 0.5% and 1%. ^abcd^: The means with different superscripts among the groups are statistically different (*p* < 0.05). (**B**) The antimicrobial properties of the chitosan (CS) films formulated using ethyl acetate (EA), ethanol (E), water (W), and a combination of ethanol-water (EW) of *B. officinalis* extracts, each of 0.5% and 1%. PC: positive control (Levofloxacin disc, 5 μg), NC: negative control (CS film). Chitosan films with aqueous extract (CS + W) added, which is not shown in the figure, did not show antimicrobial activity against any microorganisms. Moreover, none of the prepared film samples showed antimicrobial activity against *E. coli*, *S.* Typhimurium, *E. faecalis*, *K. pneumoniae*, and *C. albicans*.

The antioxidant and antimicrobial activities of CS films formulated with ethyl acetate (EA), ethanol (E), water (W), and an ethanol-water (EW) combination of *B. officinalis* extracts were evaluated (Figure 1).

All borage-supplemented films showed significantly higher antioxidant effects than CS films (*p* < 0.05). The results obtained in this study are in line with previous studies that have reported the antioxidant activity of the *B. officinalis* plant [11,13,14,25,26]. Films showed the highest antioxidant activity prepared with the ethyl acetate extract and the lowest with an aqueous extract (*p* < 0.05). Still, extracts derived from an ethanolic solution and an ethanol-water combination showed similar results (*p* > 0.05). The antioxidant capacity of plant extracts is often associated with their phenolic profile. The ethyl acetate and ethanolic extracts demonstrate more capacity for antioxidants than the aqueous extract, probably related to the polarity index of the phenolic compounds in these solvents [14].

The antimicrobial activity of CS films combined with *B. officinalis* extracts was evaluated for the microorganisms, which are species known to be human pathogens or potential foodborne pathogens. Chitosan films with aqueous extract (CS + W) added did not show antimicrobial activity against any microorganisms. Also, none of the prepared film samples showed antimicrobial activity against *E. coli*, *S.* Typhimurium, *E. faecalis*, *K. pneumoniae*, and *C. albicans* (Supplementary Materials). The maximum antimicrobial zone diameter was recorded against *S. aureus* (9.5 ± 2.1 mm) in CS films prepared with borage extract obtained with an ethanol-water mixture (Figure 1). This was followed by *B. subtilis* (9.0 ± 1.0 mm) in CS films prepared with borage extract obtained with ethyl acetate. The antimicrobial effect of the 0.5% ethyl acetate group was limited and did not provide an antimicrobial zone against *P. aeruginosa*, *E. aerogenes*, and *A. haemolyticus*. However, CS films prepared with borage extract obtained using ethanol or an ethanol-water mixture demonstrated a slight effect on these microorganisms (Figure 1). Different borage extracts have already been tested against *Salmonella* spp., *Enterobacter* spp., *E. coli*, *P. aeruginosa*, *K. pneumonia*, *Listeria monocytogenes*, *Bacillus cereus*, *B. subtilis*, and *S. aureus* [11,13]. Researchers reported the antimicrobial effect of borage extracts against *S. aureus* and *B. subtilis*, similar to the results of this study [11,13]. Furthermore, Miceli et al. (2014) have shown antimicrobial activity against some strains of *L. monocytogenes*, *Salmonella* Enteritidis, *Salmonella* Derby, and *Enterobacter sakazakii*. Although researchers have determined the in vitro antimicrobial activity of aqueous extracts of *B. officinalis*, they have not been able to confirm this effect in situ tests in fish broth, meat broth, or vegetable broth [11]. In another study, similar to the results of this study, the antimicrobial activity of the borage extracts, methanolic, ethanolic, and aqueous extracts showed the strongest, moderate, and lowest effects, respectively [13]. When compared to antioxidant and antimicrobial capacity, the most effective films for food application may be CS films made with borage extract from an ethanol-water mixture (CS + EW + 05, CS + EW + 1). Consequently, it was decided to investigate the physical properties of these films and their effects on the quality and shelf life of fresh rainbow trout.

### 3.2. Characterization of Chitosan Films Prepared with Ethanol-Water Extracts of Borago officinalis

**Figure 2:**
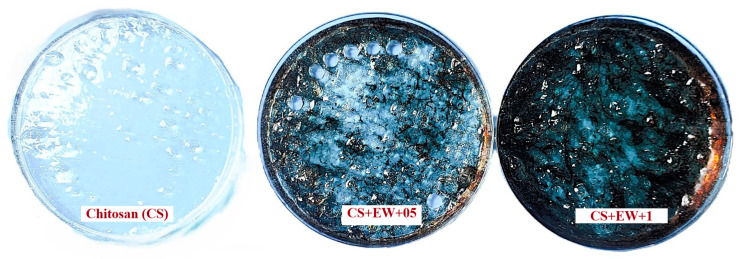
The appearance of prepared chitosan films (CS + EW + 0.5; chitosan added 0.5% *B. officinalis* ethanol-water extract; CS + EW + 1; chitosan added 1% *B. officinalis* ethanol-water extract).

The prepared chitosan-based films are shown in Figure 2.

The film’s moisture, solubility, swelling degree, and color parameters were examined. The results are summarized in Table 1.

Moisture content is an important factor in determining the physical quality of a film when treated to food products with higher water activity [27]. The addition of borage extract reduced the moisture content, solubility, and swelling degree of chitosan films (*p* < 0.05). The intermolecular interaction between chitosan molecules, water molecules, and hydroxyl/amino groups is responsible for the high moisture content in CS films. Plant extracts typically contain a high concentration of hydroxyl groups. It is possible that adding a borage extract could help make hydrogen bonds between molecules and connect hydrophilic parts in chitosan [27,28].

The darkness value of the film samples increased and the yellowness, whiteness, and browning index decreased (*p* < 0.05). Color saturation (Croma) decreased with the addition of borage to chitosan films (*p* < 0.05). These changes are due to the compounds that give color to borage extract. The Hue angle value was similar between the groups (*p* > 0.05). Values ranged from 245 to 251 degrees. This shows the blue-purple color range of the films. The total color difference (ΔE) between film samples was relatively high. The highest color change was between the CS and CS + EW + 1 groups (*p* < 0.05). The prepared film’s color change was easily visible to the naked eye (Figure 2). As known, the color change was clearly visible when the ∆E variation of the films was more than five [29].

### 3.3. Application of CS-EW Coating Films to Monitoring Rainbow Trout’s Shelf Life

**Figure 3:**
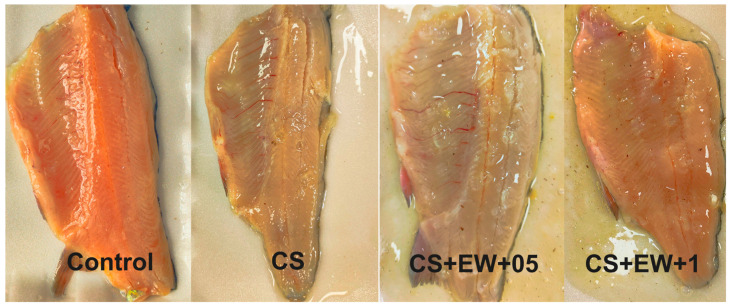
The appearance of control and chitosan coated fillet samples at first day of storage (CS; Chitosan coated sample; CS + EW + 0.5; chitosan added 0.5% *B. officinalis* ethanol-water extract; CS + EW + 1; chitosan added 1% *B. officinalis* ethanol-water extract).

The control and CS-EW film coated rainbow trout fillets are shown in Figure 3.

#### 3.3.1. Changes in Physicochemical Characteristics

After the fish samples were prepared on the first day of each repetition, the product’s moisture, ash, protein, and fat content were determined (Table 2).

**Figure 4:**
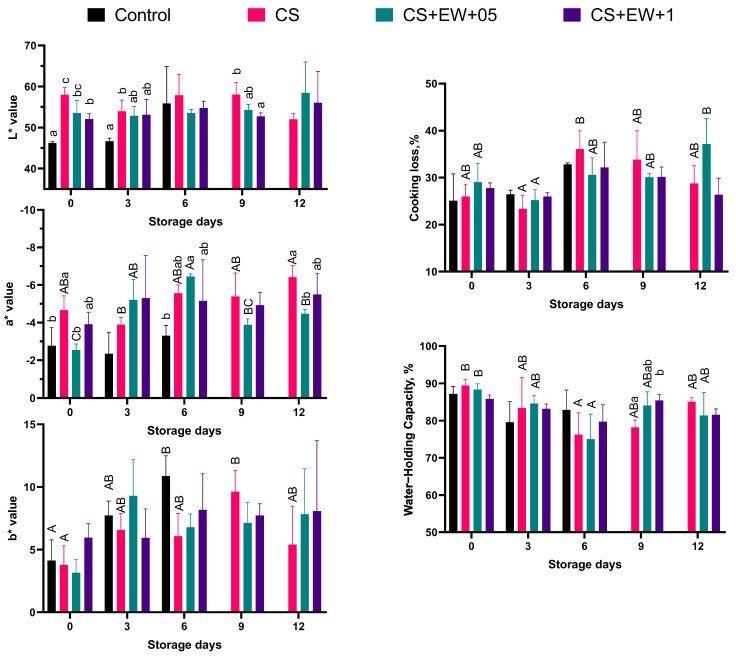
Changes in color parameters (L*, a*, b*), water-holding capacity, and cooking loss values during storage at 7 ± 1 °C for 12 days. ^ABC^: The means with different superscripts among the sampling days are statistically different; ^abc^: The means with different superscripts among the groups are statistically different (*p* < 0.05) (C: Rainbow trout fillet without films; CS: with chitosan films; CS + EW + 0.5: with chitosan added 0.5% *B. officinalis* ethanol-water extract; CS + EW + 1: with chitosan added 1% *B. officinalis* ethanol-water extract).

In addition, as a measure of meat quality on analysis days, cooking loss, water-holding capacity, and color parameters were evaluated (Figure 4).

The basic nutrient profile showed no differences between the control and chitosan groups (*p* > 0.05).

As indicated in Figure 4, initially, L* values (whiteness/darkness) were different between the control, CS, and CS + EW + 1 groups (*p* < 0.05). This difference disappeared when the control group spoiled (*p* > 0.05). As it is known, CS gives brightness to the products [30,31]. In the control group, the L* value increased with spoilage. This difference may be explained by the slime layer formed as a result of bacterial growth on the fish meat [32]. Previous studies indicate that the addition of plant extracts often changes color and decreases the transparency of the resulting CS films [9]. Similarly, on the ninth day of storage, the CS and CS + EW + 1 groups were different from each other (*p* < 0.05). Again, the a* values (redness/greenness) were different in the control and CS group at the beginning (*p* < 0.05). However, groups with added borage extract (CS + EW + 0.5, CS + EW + 1) showed values similar to those of the control group (*p* > 0.05), suggesting that the borage addition had increased the redness of the samples. Additionally, there is no significant difference in b* values (yellowness/blueness) between groups (*p* > 0.05). During the 12 days of storage, the yellowness value gradually increased over time in the control and CS groups due to protein denaturation and lipid oxidation [33,34]. However, there was no difference in borage-added groups, which means borage protected the fillet from discoloration. This was consistent with our results that borage addition reduced the yellowness index in CS films (Table 1).

Cooking loss (CL) is associated with muscle damage resulting from myofibrillar degeneration via heating, which includes the loss of water-soluble substances from muscle [35]. The CL values during storage in all groups were between 23.34 ± 2.92 and 37.15 ± 5.46, and there was no difference between groups (*p* > 0.05). Water holding capacity (WHC) is basically related to freshness. A higher WHC value indicates more freshness in meat, while a decreasing value indicates a progression of spoilage [36]. The WHC, which was initially 89.44 ± 1.55 and 88.37 ± 1.52 in the CS and CS + EW + 0.5 groups, respectively, was found to be 76.26 ± 5.85 and 75.00 ± 6.24 on the sixth day of storage (*p* < 0.05). The WHC values were not different between groups in the first six days of storage. On the ninth day of storage, the CS + EW + 1 group was relatively high compared to the CS groups (*p* < 0.05). This could suggest that the addition of borage extract (CS + EW + 1) has an impact on freshness.

#### 3.3.2. Changes in Chemical Properties

Table 3 demonstrates the variations in the pH, FFA content, TVB-N, TBA value, CS, CS + EW + 0.5, CS + EW + 1, and control samples.

Fresh rainbow trout meats have a pH of 6.28, which is within the normal pH range [37]. On the other hand, CS, CS + EW + 05, and CS + EW + 1 groups on day 0 were 6.08, 6.04, and 6.05, respectively. The initial pH decrease can be attributed to acetic acid in CS film coatings. CS is generally responsible for the low initial pH in a lot of CS-applied meat studies [15,38,39,40]. This lower pH in CS-coated samples was maintained throughout the storage period, contrary to the control sample. This trend agrees with other studies about CS-coated marine products [15,34,41]. The observed increase in pH in control samples throughout the storage period may be attributed to the rise in total volatile basic nitrogen (TVB-N) compounds, which is likely a consequence of proteolytic activities by microorganisms or endogenous enzymes. [22]. Also, adding borage extract to CS-coated samples did not affect the pH value of the fillets (*p* > 0.05). This is important for the stability of the coating material during the storage period [42].

TVB-N is a critical indicator of seafood freshness, exhibiting a strong correlation with pH levels and the decarboxylase activity of bacterial spoilage. The suggested upper limit of tolerability for rainbow trout meat is 25 mg/100 g TVB-N [16]. In the current research, only the control group exceeded the limit on storage day six. However, CS groups exceeded this limit on day twelve. The TVB-N level of all samples increased during 7 ± 1 °C storage because of the microbiological degradation of protein by producing alkaline substances such as ammonia and tri-methylamine [43]. The TVB-N in CS and borage-supplemented CS groups can be attributed to the impact of antimicrobial compounds in reducing the proliferation of spoilage microorganisms and consequently inhibiting lipid oxidation and the degradation of meat proteins. The present study is in accordance with the studies by Raeisi et al. (2020), Feng et al. (2016), Rezaeifar et al. (2020), and Ebadi et al. (2019) [16,43,44,45].

The development of lipid hydrolysis was studied by measuring free fatty acids (FFA), which are products of triacylglycerols formed through enzyme-mediated hydrolysis. In general, enzymes within the meat or bacteria can cause hydrolytic oxidation in the fillets, leading to an overall increase. Moreover, protein-triacylglycerol interactions may explain decreased FFA content [46]. According to the results obtained from this study, TVB-N and FFA values were developed with similar trends. On treatment day, the FFA levels of Control, CS, CS + EW + 0.5, and CS + EW + 1 samples were 67.99 ± 9.70, 66.96 ± 8.80, 74.15 ± 18.30, 50.60 ± 21.49, respectively. In the CS groups supplemented with borage extract, the standard deviation values were high, while the FFA average was low. This may be attributed to the dynamic and interactive chemical properties of borage compounds with the samples. Some researchers indicated that CS coating has positive effects on reducing FFA [16,47,48,49,50], confirming the current research. However, it cannot be said that adding borage extract affects the FFA value in the CS-coated fillets.

The TBA value indicates oxidative damage to lipids that detect malondialdehyde (MDA) content. The advised maximum acceptable limit for MDA concentration is 3 mg/kg in high-quality fish [51]. On the 12th day of storage, the CS group exceeded the limit, and all groups approached the limit value. The initial MDA level for control and treatment groups ranged from 0.55 ± 0.07–0.65 ± 0.15 mg/kg of fillet, inconsistent with other researchers [22,44,52,53]. On the third day of storage, groups supplemented with borage extract showed lower MDA levels than the control (*p* < 0.05). Then, at six days of storage, all chitosan groups had lower MDA content than the control (*p* < 0.05). Additionally, the 1% borage extract-supplemented group had lower MDA values than the chitosan group on the 9th and 12th days of storage (*p* < 0.05). As the storage period progressed, the MDA level in all CS groups remained lower than in the control group. This indicates that the CS and the CS with borage extract demonstrated an antioxidant effect compared to the control group. TBA results confirm our results of radical scavenging activities on DPPH applied to film samples (Figure 1). Oxygen is needed for lipid oxidation. It is known that CS film has an important effect on meat oxidation because of oxygen barrier properties [54]. According to Hasdemir et al. (2023) [55], supplementation of fish oil with 1% *Borago officinalis* protects fish oil and prevents oxidation, which is parallel with the results of the present research.

#### 3.3.3. Changes in Microbiological Properties

**Figure 5:**
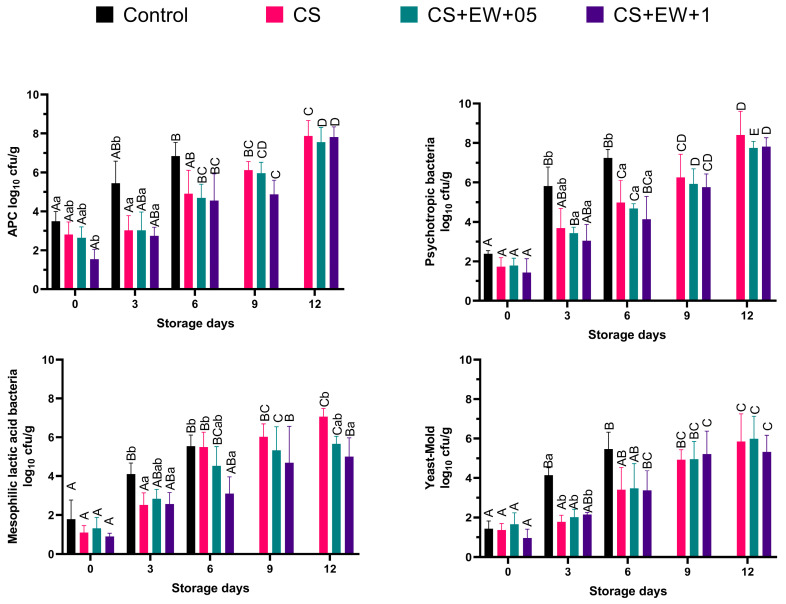
Changes in Aerobic plate counts (APC), psychrotrophic bacteria, mesophilic lactic acid bacteria (LAB), and yeast-mold (YM) counts of experimental groups during storage at 7 ± 1 °C for 12 days. ^ABCDE^: The means with different superscripts among the sampling days are statistically different; ^ab^: The means with different superscripts among the groups are statistically different (*p* < 0.05). (C: Rainbow trout fillet without films; CS: with chitosan films; CS + EW + 0.5: with chitosan added 0.5% *Borago officinalis* ethanol-water extract; CS + EW + 1: with chitosan added 1% *Borago officinalis* ethanol-water extract).

The changes in aerobic plate counts (APC), psychrotrophic bacteria (PB), mesophilic lactic acid bacteria (LAB), and yeast-mould (YM) counts of experimental groups during storage at 7 ± 1 °C for 12 days are presented in Figure 5.

The initial counts of APC, PB, LAB, and YM in samples were between 3.49–1.54, 2.38–1.43, 1.79–0.90, and 1.64–0.96 log cfu/g, respectively, which indicates good quality fresh fish. It was observed that all tested microorganism groups of all groups increased gradually during the storage period (*p* < 0.05). As expected, the spoil rate of the control group was significantly higher than that of the CS groups in microbiologically (*p* < 0.05). On day 6, the APC and PB counts of the control group were 6.84 and 7.24 log cfu/g, respectively. It was close to the suggested limit of 7.0 log cfu/g for meat by the “International Commission on Microbiological Specifications for Foods (ICMSF)”. In addition, CS groups exceeded this limit on the 12th day of storage. The literature clearly states that CS coatings extend the shelf life of foods and have an antimicrobial effect [8,9,54,56]. The antimicrobial effect present in this study was due to chitosan. Although there is evidence for the antimicrobial effect of borage extracts [11,13], it cannot be said that 1% ethanol-water extract added to CS has an antimicrobial effect on fish fillets (*p* > 0.05). The microbiological results nearly confirm our results of antimicrobial activities in film samples (Figure 1). Furthermore, the study by Miceli et al. (2014) [11], which found that aqueous extracts of *B. officinalis* demonstrated antimicrobial activity in vitro tests, could not confirm this effect in situ tests in fish broth.

## 4. Conclusions

This research aimed to apply chitosan-based films enriched with *Borago officinalis* extracts for active and green packaging of fresh Rainbow trout fillets. The antioxidant activity of borage extracts demonstrated the strongest effects with ethyl acetate, modest effects with ethanol-water, and the lowest effects with water. The results demonstrated that the chitosan films enriched with 1% *Borago officinalis* ethanol-water extract can be applied to fish fillets. The films extend the shelf life of rainbow trout fillets at 7 ± 1 °C by about six days. Also, Borage extract protected rainbow trout fillets from discoloration and oxidation. It can be thought that the films have a sufficient antioxidant effect but a slightly antimicrobial effect. Further interventions and studies could focus on improving the antibacterial effectiveness of films. Consequently, chitosan films with borage extract, developed through this research, may provide enhanced stability and efficacy in the shelf life of fast perishable food products.

## Figures and Tables

**Table 1:** The moisture, solubility, swelling degree, and color parameters of chitosan films.

	CS	CS + EW + 05	CS + EW + 1
**Moisture (%)**	31.18 ± 1.95 ^b^	28.65 ± 1.99 ^b^	22.36 ± 2.14 ^a^
**Solubility (%)**	31.07 ± 2.27	25.53 ± 4.40	23.10 ± 2.59
**Swelling degree (%)**	53.08 ± 2.89 ^b^	49.33 ± 1.29 ^ab^	45.27 ± 2.03 ^a^
L*	92.17 ± 1.96 ^a^	43.35 ± 1.86 ^b^	23.28 ± 4.15 ^c^
a*	−5.23 ± 0.67	−4.64 ± 0.92	−3.54 ± 2.08
b*	−11.34 ± 0.97 ^b^	−13.90 ± 0.69 ^a^	−7.28 ± 1.11 ^c^
**Yellowness Index (YI)**	−17.60 ± 1.84 ^b^	−45.80 ± 0.90 ^a^	−44.87 ± 3.07 ^a^
**Whiteness Index (WI)**	85.23 ± 1.93 ^a^	41.48 ± 1.73 ^b^	22.82 ± 3.93 ^c^
**Browning Index (BI)**	−5.30 ± 0.70 ^a^	−10.89 ± 1.96 ^ab^	−13.60 ± 4.71 ^b^
Croma (C*)	12.49 ± 1.09 ^b^	14.68 ± 0.50 ^b^	8.19 ± 1.85 ^a^
Hue Angle (h*)	245.27 ± 2.01	251.50 ± 4.06	245.64 ± 10.50
**ΔE**	**CS&CS + EW + 05** 48.91 ± 1.19 ^b^	**CS&CS + EW + 1** 69.08 ± 3.00 ^a^	**CS + EW + 05&CS + EW + 1** 21.16 ± 4.16 ^c^

(CS: without B. officinalis extract; CS + EW + 0.5: chitosan added 0.5% B. officinalis ethanol-water extract; CS + EW + 1: chitosan added 1% B. officinalis ethanol-water extract). ^abc^: The means with different superscripts among the groups are significantly different (*p* < 0.05).

**Table 2:** Moisture, crude protein, crude fat, and ash content in the fresh rainbow trout fillet samples (g/100 g).

	Control	CS	CS + EW + 05	CS + EW + 1
**Moisture**	69.54 ± 6.22	67.61 ± 1.15	64.91 ± 6.69	68.60 ± 1.10
**Protein**	21.10 ± 4.31	22.90 ± 4.97	21.26 ± 9.03	23.73 ± 2.37
**Fat**	5.22 ± 3.37	6.06 ± 3.01	5.38 ± 1.34	4.90 ± 2.53
**Ash**	1.34 ± 0.16	2.37 ± 1.93	1.14 ± 0.24	1.42 ± 0.24

(Control: Fresh fillet without films; CS: Chitosan films; CS + EW + 0.5: chitosan added 0.5% *B. officinalis* ethanol-water extract; CS + EW + 1: chitosan added 1% *B. officinalis* ethanol-water extract).

**Table 3:** Changes in pH, FFA, TVB-N, and TBA value of rainbow trout samples during storage at 7 ± 1 °C for 12 days.

pH			Control	CS	CS + EW + 05	CS + EW + 1
**Storage days**		**0**	6.28 ± 0.15 ^Ab^	6.08 ± 0.15 ^a^	6.04 ± 0.15 ^a^	6.05 ± 0.25 ^Aa^
		**3**	6.39 ± 0.06 ^bAB^	6.09 ± 0.07 ^a^	6.14 ± 0.15 ^a^	6.19 ± 0.07 ^ABa^
		**6**	6.43 ± 0.06 ^Bb^	6.17 ± 0.14 ^a^	6.19 ± 0.04 ^a^	6.14 ± 0.05 ^ABa^
		**9**		6.25 ± 0.10	6.16 ± 0.05	6.29 ± 0.11 ^AB^
		**12**		6.14 ± 0.07	6.20 ± 0.15	6.18 ± 0.07 ^B^
**TVB-N (mg/100 g)**						
**Storage days**	**0**		20.43 ± 2.16 ^Aa^	17.36 ± 2.19 ^Aab^	16.85 ± 0.82 ^Ab^	16.64 ± 0.31 ^Ab^
	**3**		21.37 ± 4.29 ^A^	18.08 ± 1.17 ^A^	18.80 ± 2.65 ^A^	18.21 ± 0.67 ^A^
	**6**		35.46 ± 5.93 ^Ba^	20.61 ± 2.39 ^Ab^	19.59 ± 3.44 ^Ab^	19.92 ± 2.46 ^Ab^
	**9**			24.07 ± 2.49 ^AB^	21.03 ± 1.29 ^AB^	20.54 ± 1.36 ^AB^
	**12**			28.41 ± 3.89 ^B^	26.56 ± 1.36 ^B^	24.81 ± 2.41 ^B^
**FFA (mg/100 g)**						
**Storage days**		**0**	67.99 ± 9.70 ^A^	66.96 ± 8.80 ^A^	74.15 ± 18.30 ^AB^	50.60 ± 21.49 ^A^
		**3**	67.99 ± 25.78 ^A^	64.96 ± 8.14 ^A^	94.03 ± 5.87 ^AB^	76.03 ± 3.03 ^A^
		**6**	113.57 ± 15.24 ^Ba^	79.10 ± 5.99 ^Aa^	88.07 ± 5.08 ^ABa^	84.16 ± 4.75 ^ABb^
		**9**		122.53 ± 14.16 ^B^	102.99 ± 33.89 ^AB^	91.51 ± 19.36 ^AB^
		**12**		125.58 ± 13.23 ^B^	126.71 ± 18.21 ^B^	129.71 ± 28.65 ^B^
**TBA (mg malonaldehyde/kg)**						
**Storage days**		**0**	0.55 ± 0.07 ^A^	0.64 ± 0.19 ^A^	0.65 ± 0.15 ^A^	0.57 ± 0.20 ^A^
		**3**	1.17 ± 0.13 ^Ba^	0.87 ± 0.06 ^Aab^	0.84 ± 0.11 ^ABb^	0.66 ± 0.15 ^Ab^
		**6**	2.84 ± 0.14 ^Ca^	1.50 ± 0.18 ^Bb^	1.21 ± 0.20 ^Bb^	1.17 ± 0.11 ^Bb^
		**9**		2.48 ± 0.30 ^Ca^	1.87 ± 0.27 ^Cab^	1.75 ± 0.16 ^Cb^
		**12**		3.25 ± 0.21 ^Da^	2.76 ± 0.09 ^Db^	2.58 ± 0.23 ^Db^

^ABCD^: The means with different superscripts among the storage days are significantly different; ^ab^: The means with different superscripts among the groups (Control: Rainbow trout fillet without films; CS: with chitosan films; CS + EW + 0.5: with chitosan added 0.5% *B. officinalis* ethanol-water extract; CS + EW + 1: with chitosan added 1% *B. officinalis* ethanol-water extract).

## Data Availability

The original contributions presented in this study are included in the article/Supplementary Materials. Further inquiries can be directed to the corresponding author.

## Supplementary Materials

The following supporting information can be downloaded at: https://www.mdpi.com/article/10.3390/foods14040639/s1, Figure S1: Antimicrobial activity tests against prepared film samples.
