# Supplementary material for: Applying Chitosan-Based Films Enriched with Borago officinalis Extract for Active and Green Packaging of Fresh Rainbow Trout Fillets
Source: Foods. 2025 Feb 14;14(4):639. doi: 10.3390/foods14040639 (PMC11854841; doi:10.3390/foods14040639)
Supplement: Supplementary file 1 [file foods-14-00639-s001.zip › foods-3457000-supplementary.pdf]

S. aureus

No	Tests	Zone diameter (mm)
**1**	**CS+EA (%1)**	**8.3±0.5**
**2**	**CS+EA (%0.5)**	**7±0.0**
3	CS+E (%1)	Not available.
4	CS+E (%0.5)	Not available.
**5**	**CS+EW(%1)**	**9.5±2.1**
**6**	**CS+EW (%0.5)**	**8.3±1.5**
7	Water (%1)	Not available.
8	Water (%0.5)	Not available.
NC	Negative control	Not available.
PC	Levofloxacine	27.8±1.3

Chitosan (CS) films formulated using ethyl acetate (EA), ethanol (E), water (W), and a combination of ethanol-water (EW) of B. officinalis extracts, each of 0.5% and 1%. PC: positive control (Levofloxacin disc, 5 µg), NC: negative control (CS film).

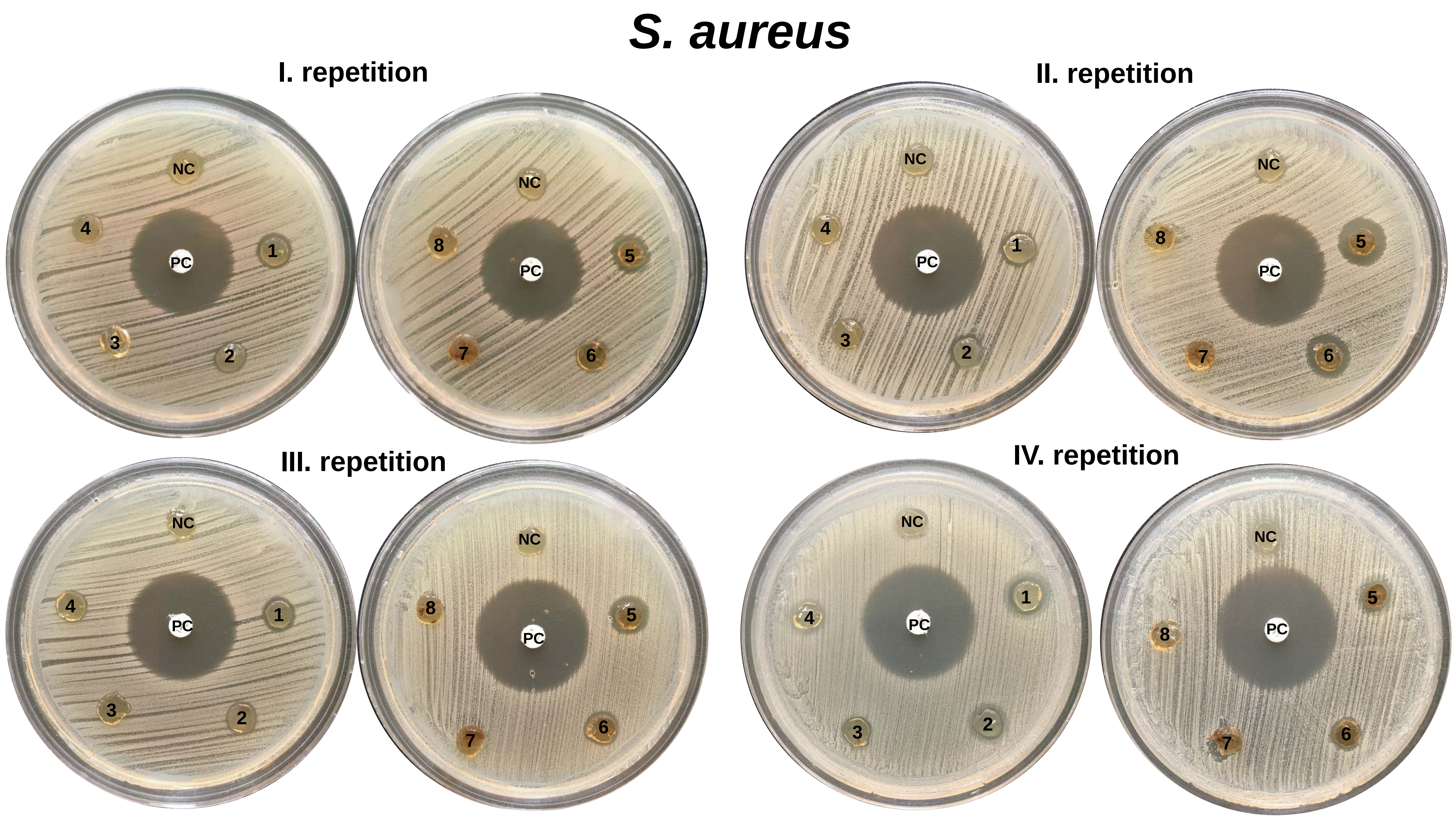
S. aureus
I. repetition
NC
4
1
PC
3
2
NC
8
5
PC
7
6
II. repetition
NC
4
1
PC
3
2
NC
8
5
PC
7
6
III. repetition
NC
4
1
PC
3
2
NC
8
5
PC
7
6
IV. repetition
NC
1
4
PC
3
2
NC
5
8
PC
7
6

E. faecalis

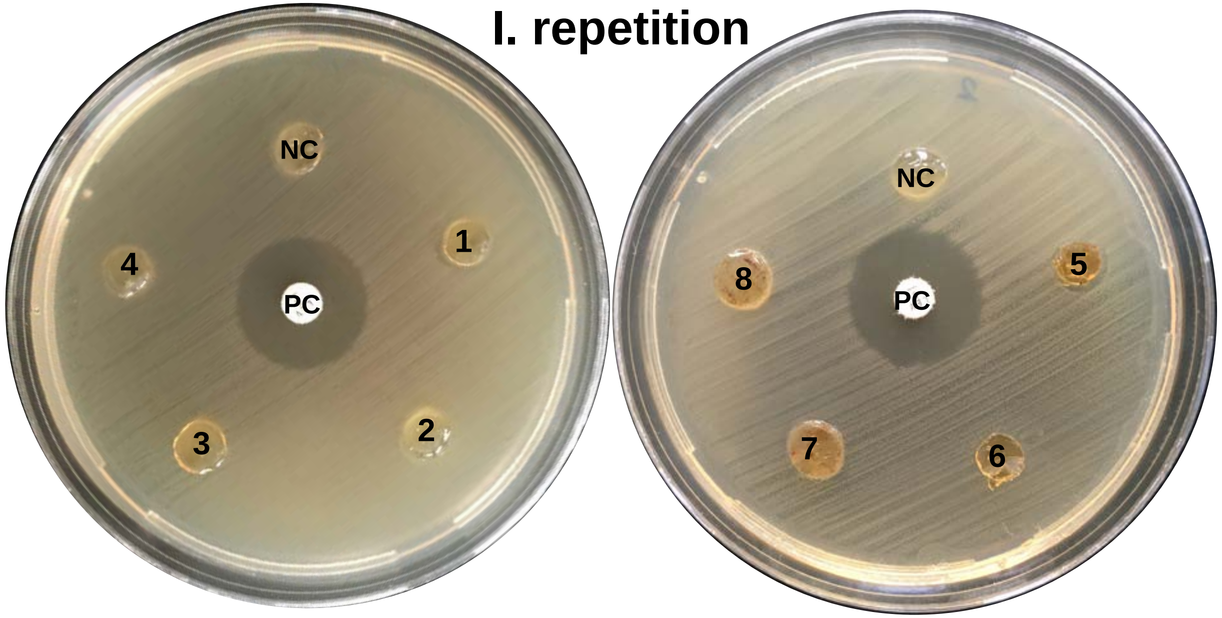

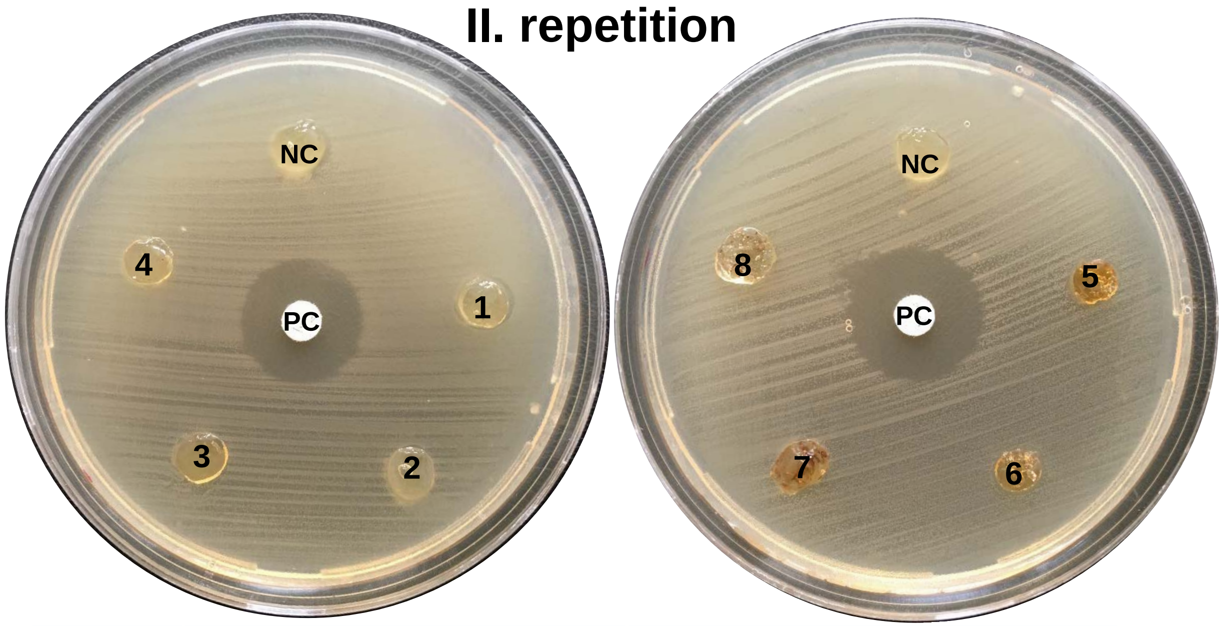

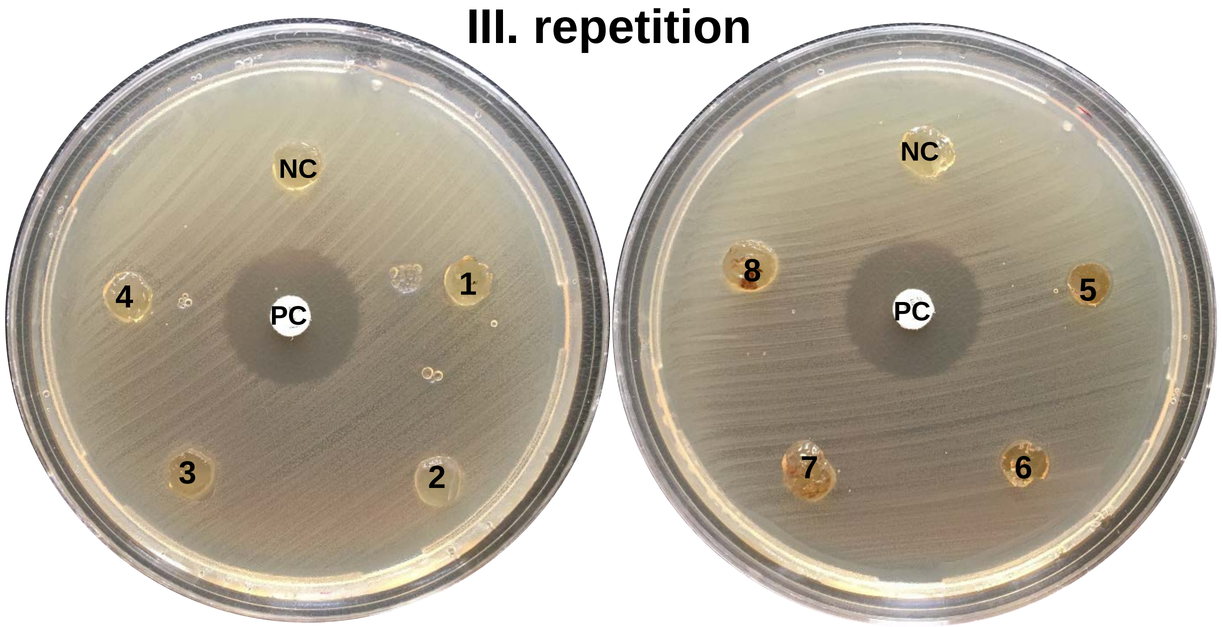

No	Tests	Zone diameter (mm)
**1**	**CS+EA (%1)**	Not available.
**2**	**CS+EA (%0.5)**	Not available.
3	CS+E (%1)	Not available.
4	CS+E (%0.5)	Not available.
**5**	**CS+EW(%1)**	Not available.
**6**	**CS+EW (%0.5)**	Not available.
7	Water (%1)	Not available.
8	Water (%0.5)	Not available.
NC	Negative control	Not available.
PC	levofloxacine	19.3±0.6

B. subtilis

I. repetition

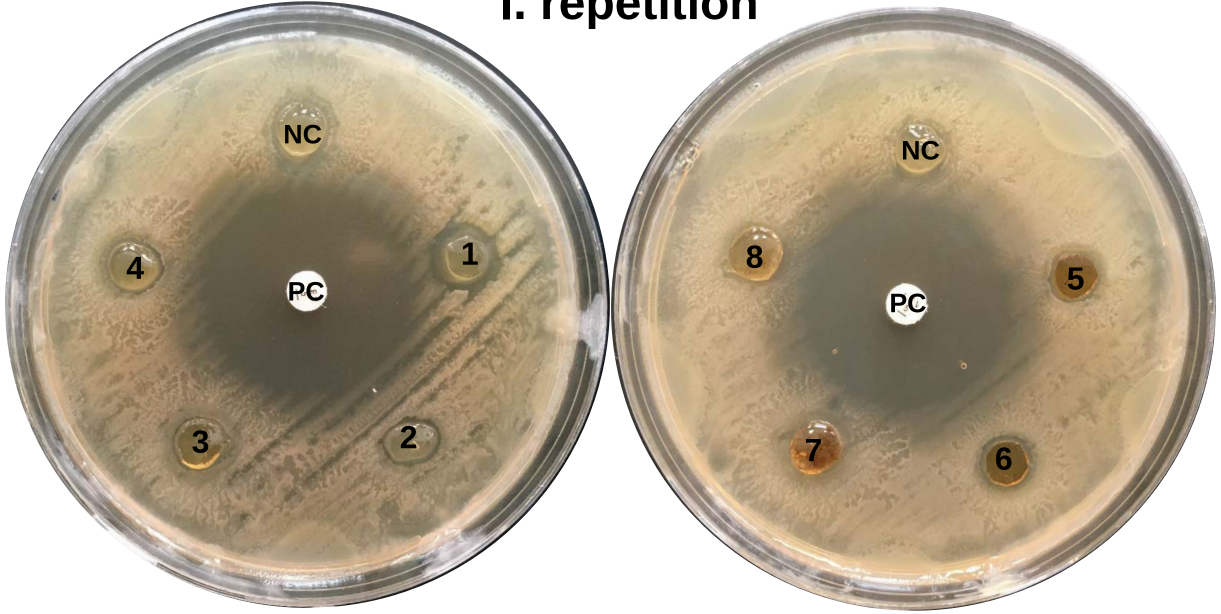

II. repetition

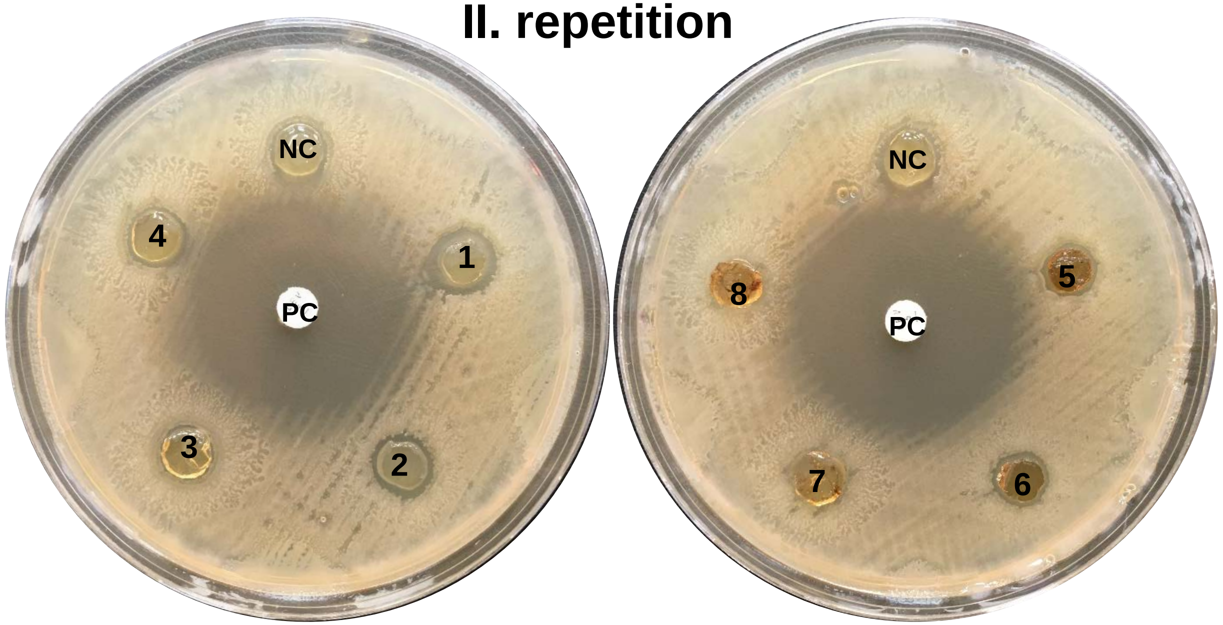

III. repetition

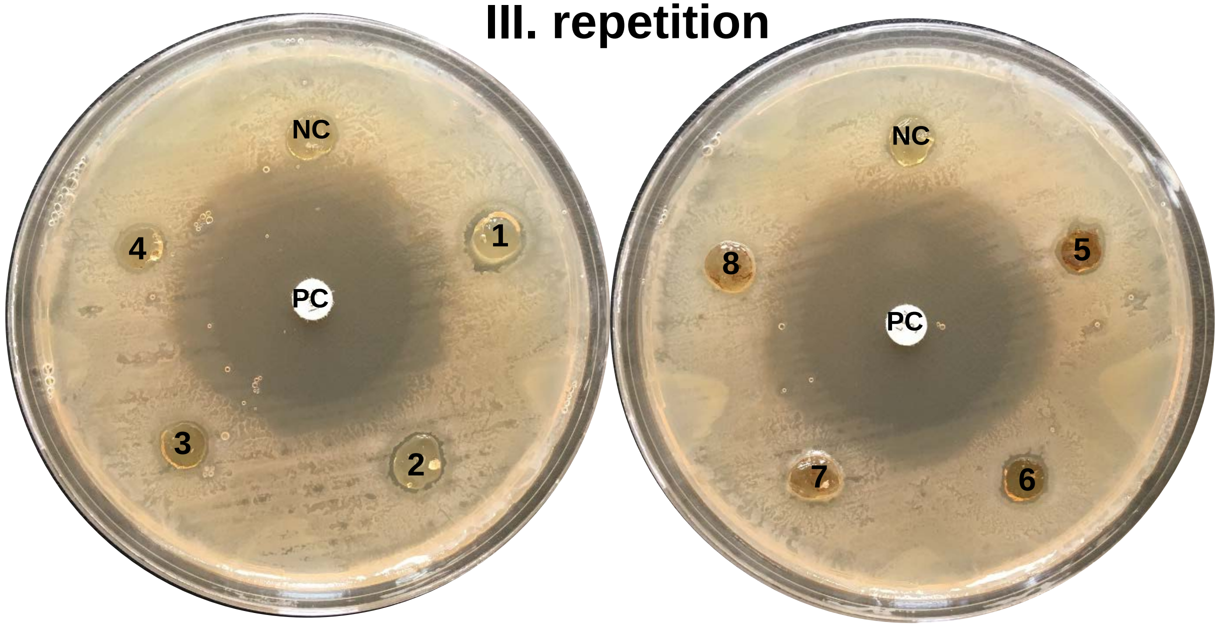

No	Tests	Zone diameter (mm)
**1**	**CS+EA (%1)**	**9±1.0**
**2**	**CS+EA (%0.5)**	**7.3±0.6**
3	CS+E (%1)	6.7±0.6
4	CS+E (%0.5)	6.7±0.6
**5**	**CS+EW(%1)**	6.7±0.6
**6**	**CS+EW (%0.5)**	6.7±0.6
7	Water (%1)	Not available.
8	Water (%0.5)	Not available.
NC	Negative control	6.7±0.6
PC	levofloxacine	32.7±0.6

E. coli

I. repetition

II. repetition

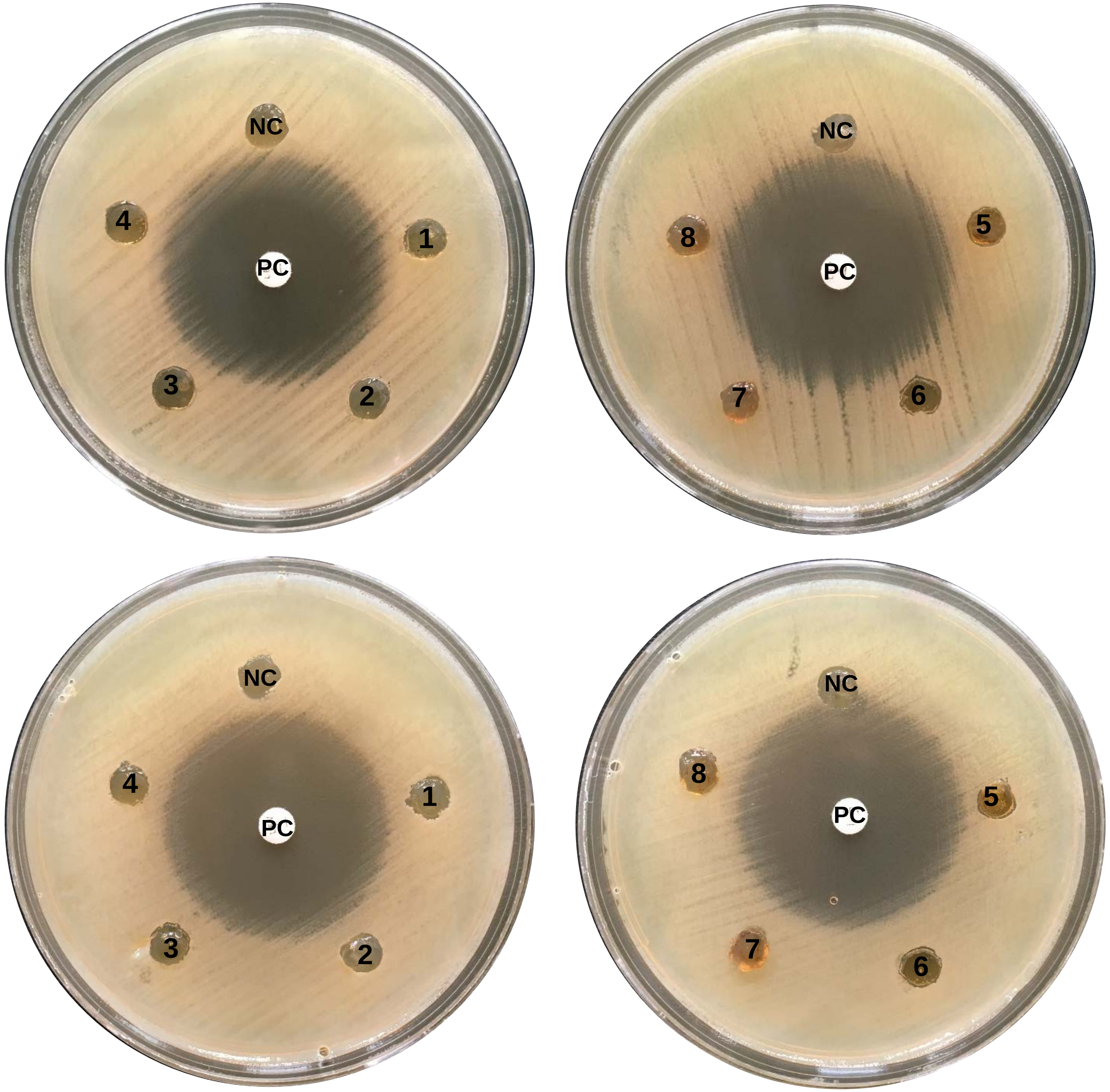

No	Tests	Zone diameter (mm)
**1**	**CS+EA (%1)**	Not available.
**2**	**CS+EA (%0.5)**	Not available.
3	CS+E (%1)	Not available.
4	CS+E (%0.5)	Not available.
**5**	**CS+EW(%1)**	Not available.
**6**	**CS+EW (%0.5)**	Not available.
7	Water (%1)	Not available.
8	Water (%0.5)	Not available.
NC	Negative control	Not available.
PC	levofloxacine	29±0.0

S. Typhimurium

I. repetition

II. repetition

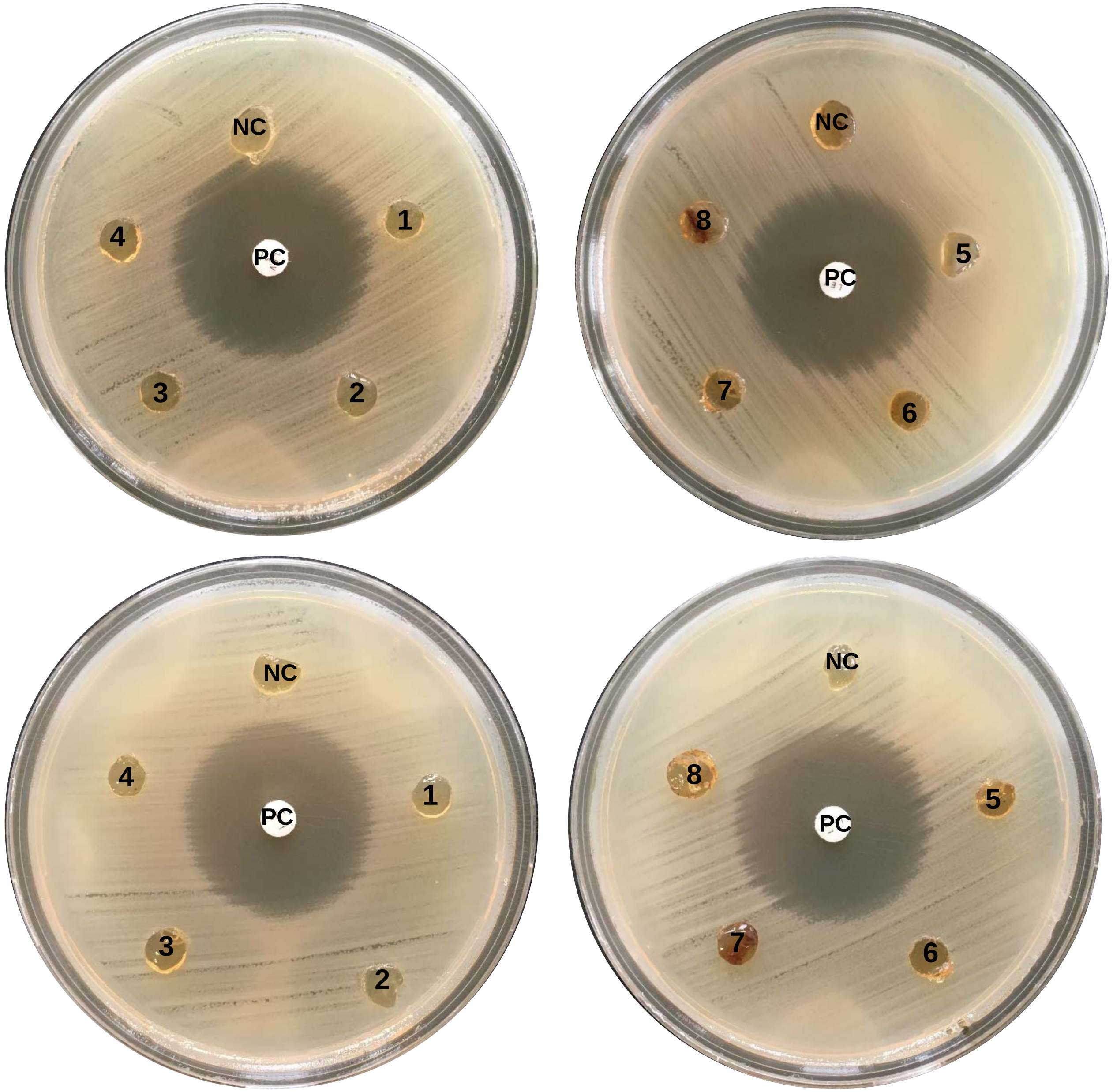

No	Test Adı	Zone diameter (mm)
No	Tests	Not available.
**1**	**CS+EA (%1)**	Not available.
**2**	**CS+EA (%0.5)**	Not available.
3	CS+E (%1)	Not available.
4	CS+E (%0.5)	Not available.
**5**	**CS+EW(%1)**	Not available.
**6**	**CS+EW (%0.5)**	Not available.
7	Water (%1)	Not available.
8	Water (%0.5)	Not available.
PC	levofloxacine	29±0.0

E. aerogenes

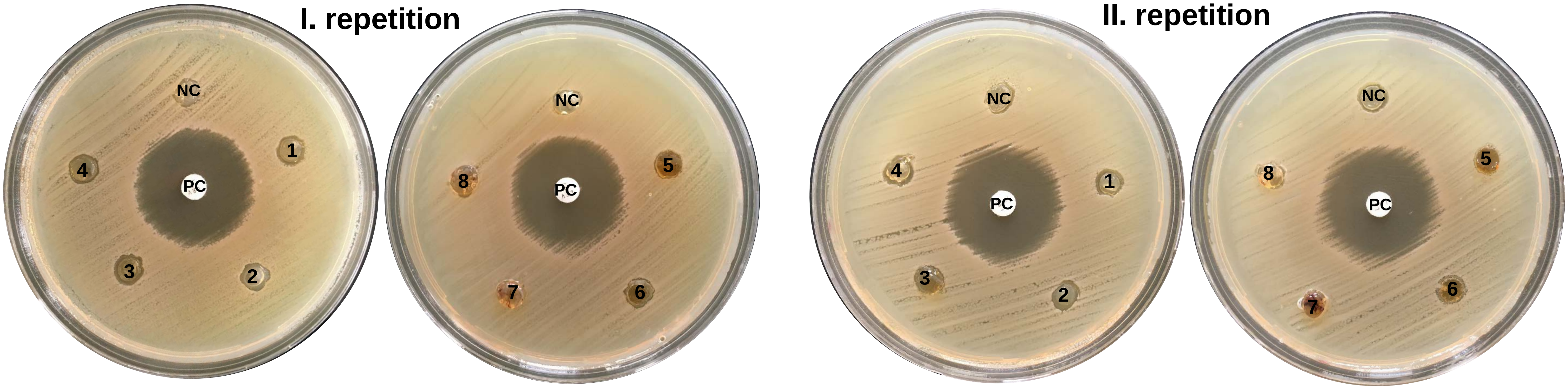

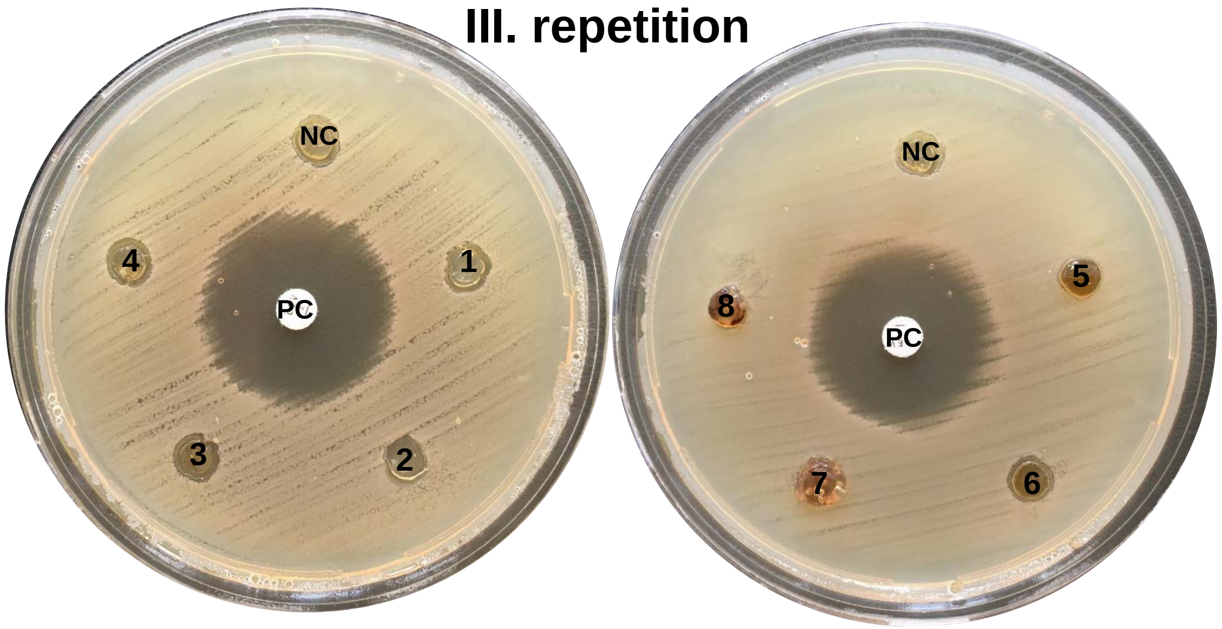

No	Tests	Zone diameter (mm)
**1**	**CS+EA (%1)**	6.7±0.6
**2**	**CS+EA (%0.5)**	Not available.
3	CS+E (%1)	6.7±0.6
4	CS+E (%0.5)	6.7±0.6
**5**	**CS+EW(%1)**	Not available.
**6**	**CS+EW (%0.5)**	Not available.
7	Water (%1)	Not available.
8	Water (%0.5)	Not available.
NC	Negative control	6.7±0.6
PC	levofloxacine	25.6±0.6

K. pneumoniae

I. repetition

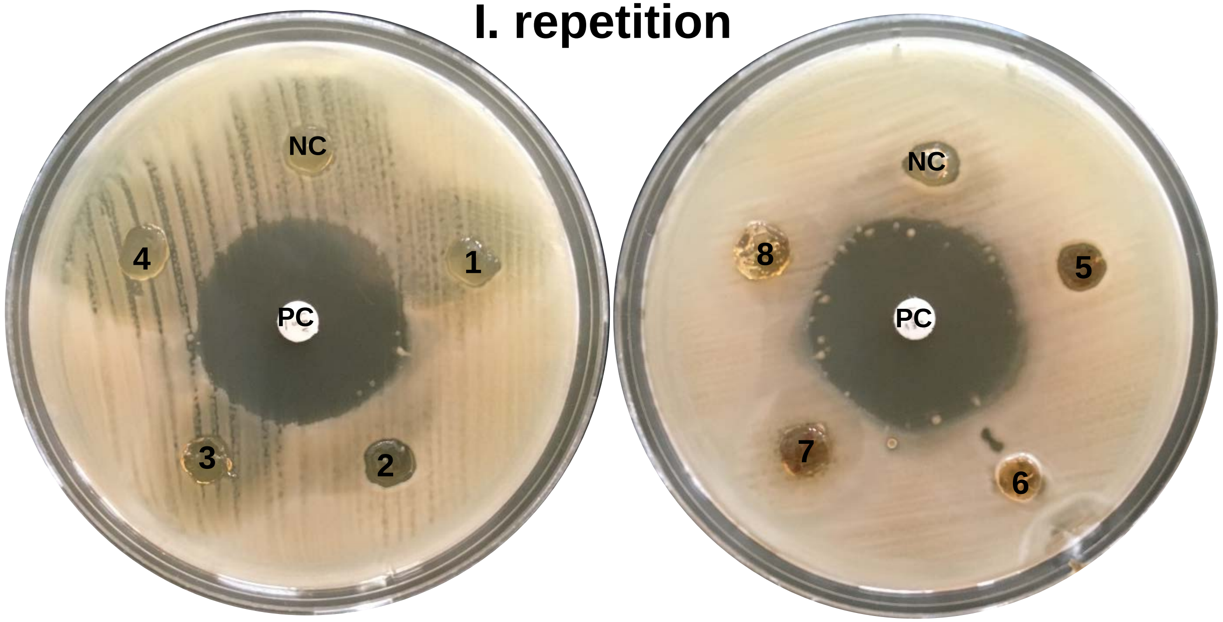

II. repetition

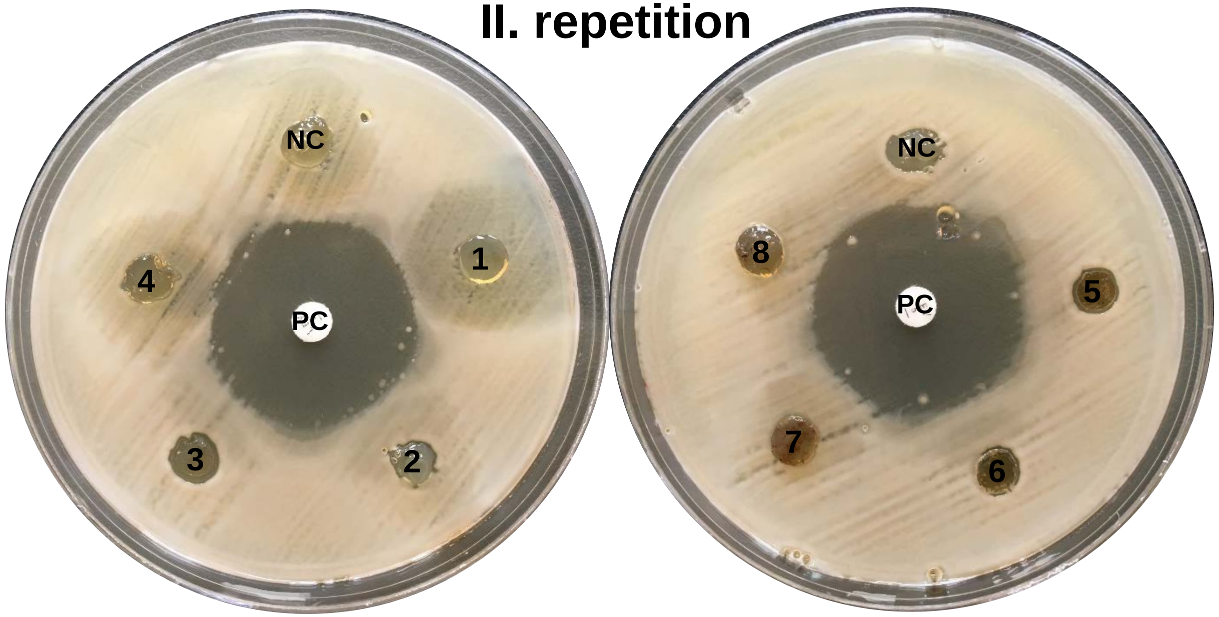

III. repetition

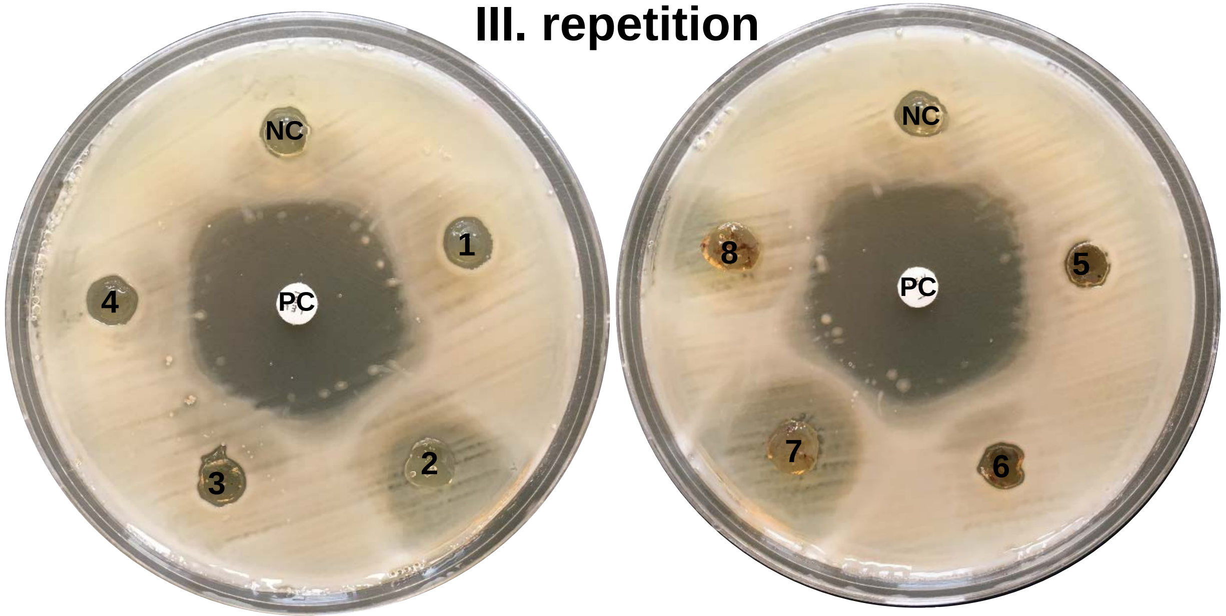

No	Tests	Zone diameter (mm)
**1**	**CS+EA (%1)**	Not available.
**2**	**CS+EA (%0.5)**	Not available.
3	CS+E (%1)	Not available.
4	CS+E (%0.5)	Not available.
**5**	**CS+EW(%1)**	Not available.
**6**	**CS+EW (%0.5)**	Not available.
7	Water (%1)	Not available.
8	Water (%0.5)	Not available.
NC	Negative control	Not available.
PC	levofloxacine	28.6±1.5

P. aeruginosa

I. repetition

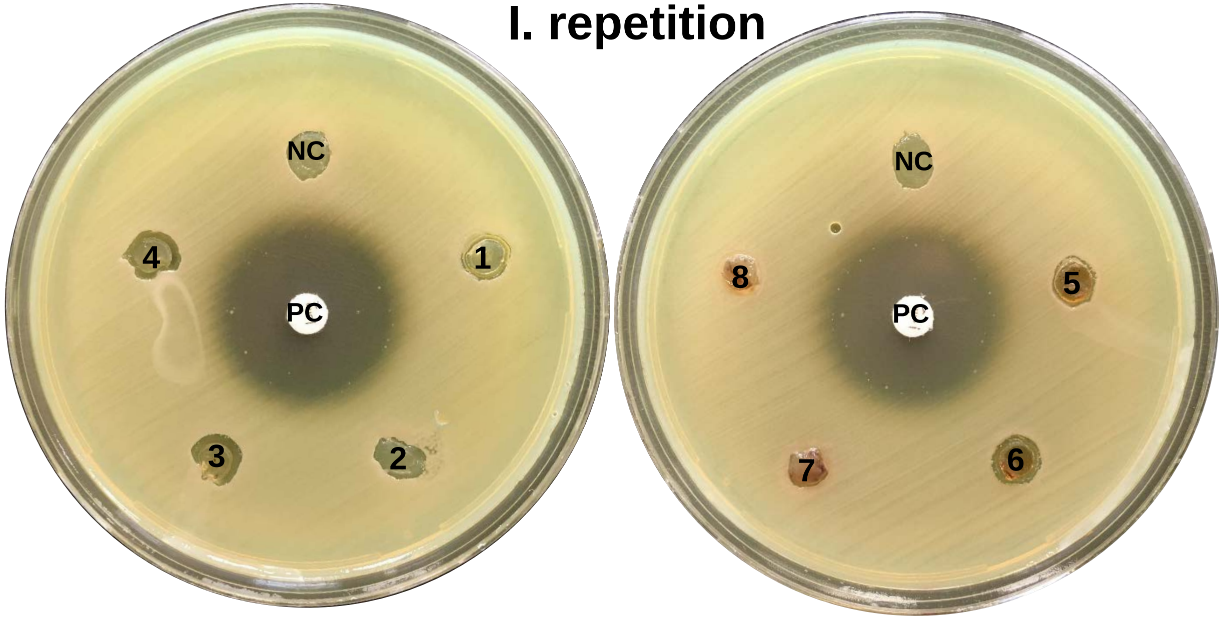

II. repetition

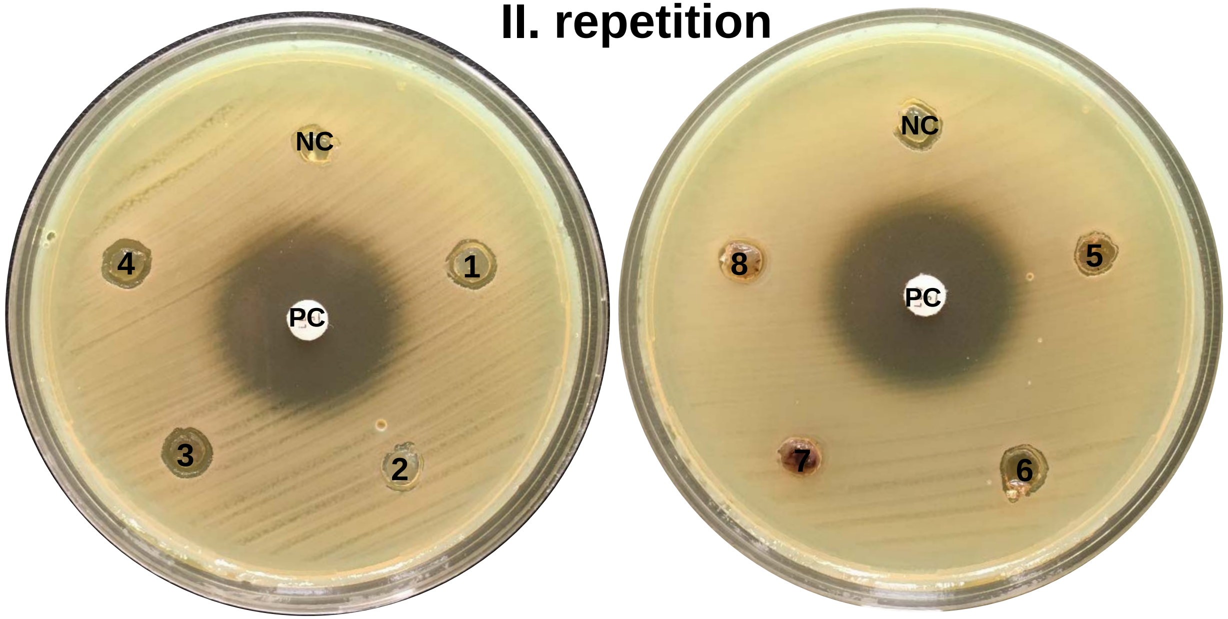

III. repetition

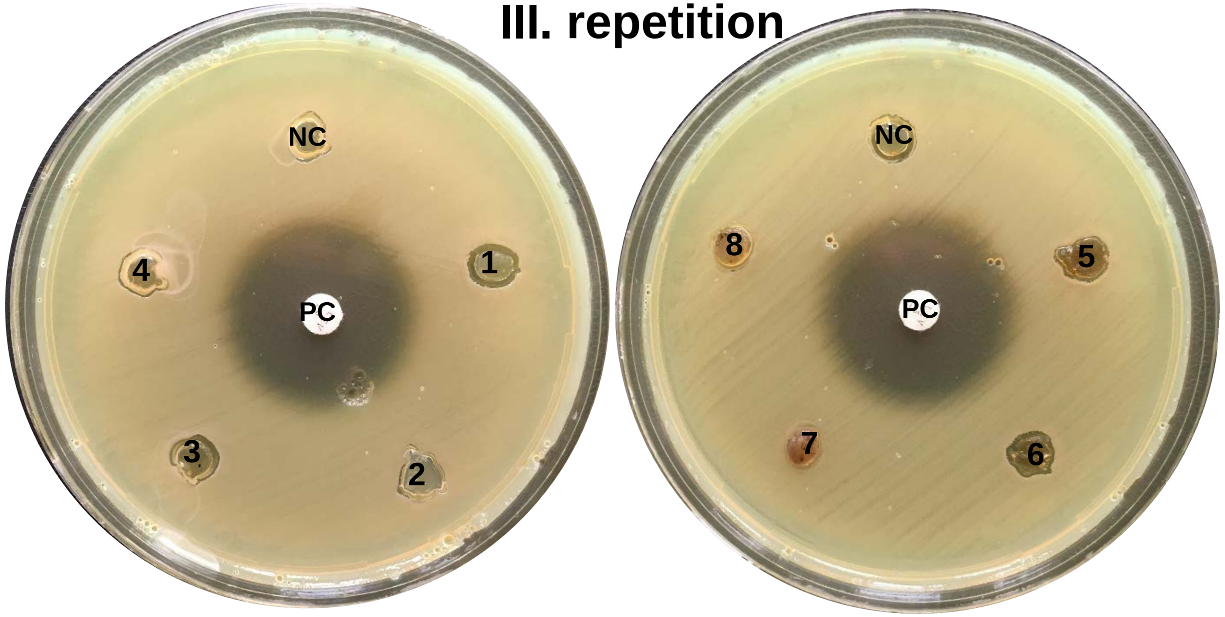

No	Tests	Zone diameter (mm)
**1**	**CS+EA (%1)**	6.3±0.6
**2**	**CS+EA (%0.5)**	Not available.
3	CS+E (%1)	**6.7±0.6**
4	CS+E (%0.5)	**6.7±0.6**
**5**	**CS+EW(%1)**	6.3±0.6
**6**	**CS+EW (%0.5)**	6.3±0.6
7	Water (%1)	Not available.
8	Water (%0.5)	Not available.
NC	Negative control	6.3±0.6
PC	levofloxacine	21.3±0.6

A. haemolyticus

I. repetition

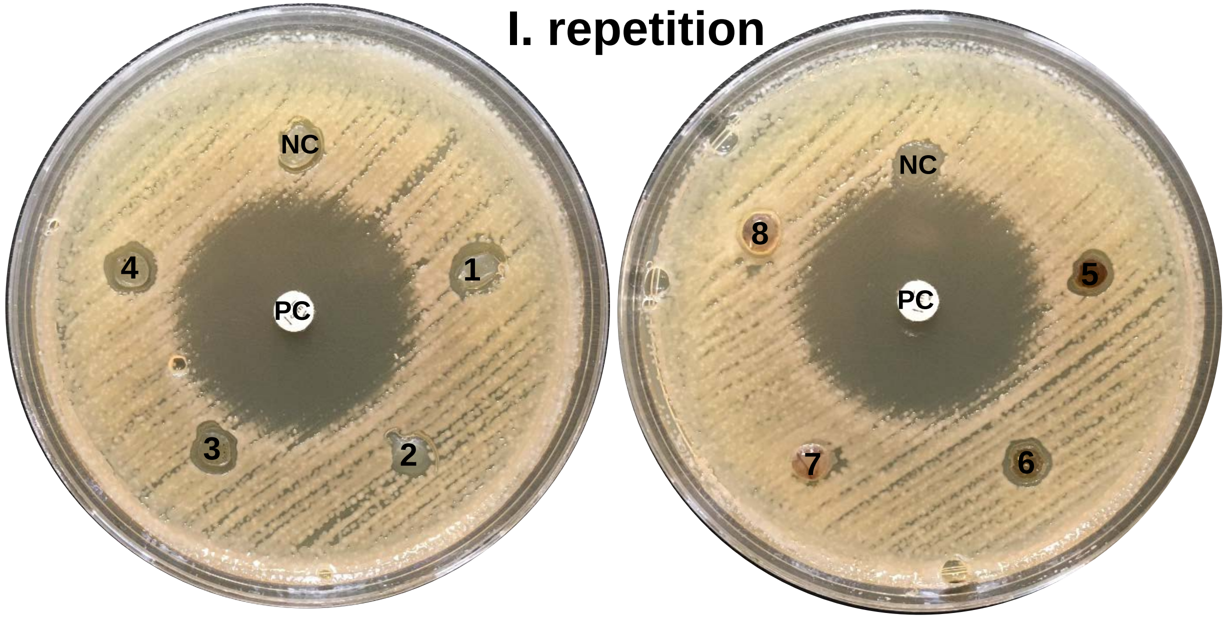

II. repetition

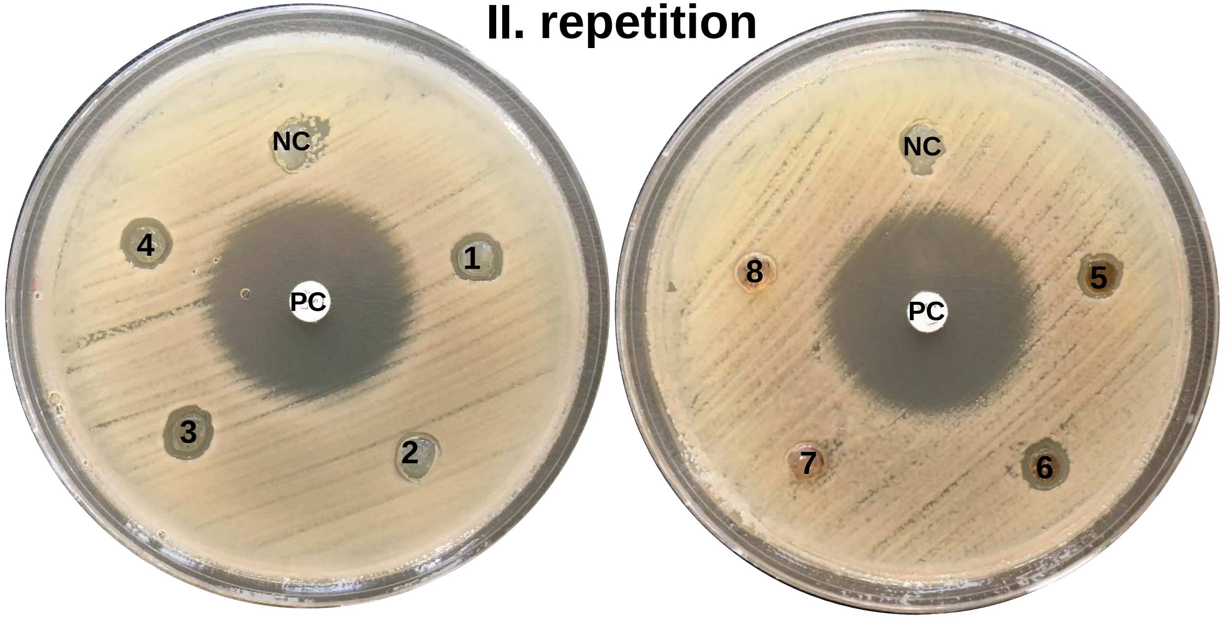

III. repetition

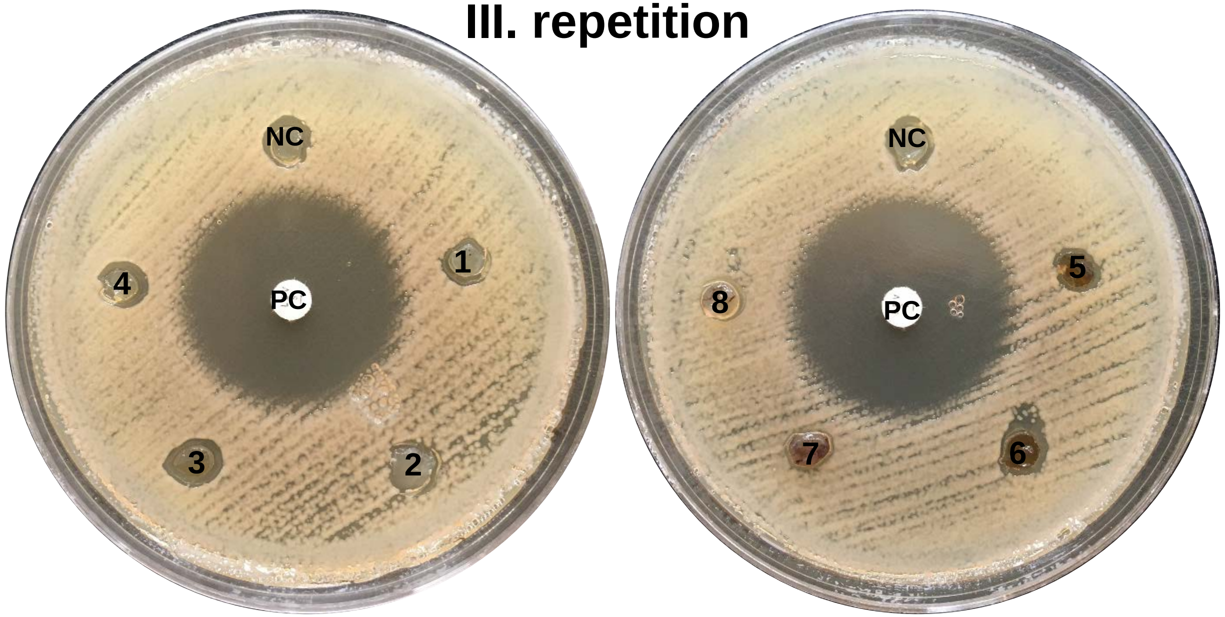

No	Tests	Zone diameter (mm)
**1**	**CS+EA (%1)**	6.7±0.6
**2**	**CS+EA (%0.5)**	Not available.
3	CS+E (%1)	**7±0.0**
4	CS+E (%0.5)	**7±0.0**
**5**	**CS+EW(%1)**	6.7±0.6
**6**	**CS+EW (%0.5)**	6.7±0.6
7	Water (%1)	Not available.
8	Water (%0.5)	Not available.
NC	Negative control	6.7±0.6
PC	levofloxacine	30.3±1.2

C. albicans

I. repetition

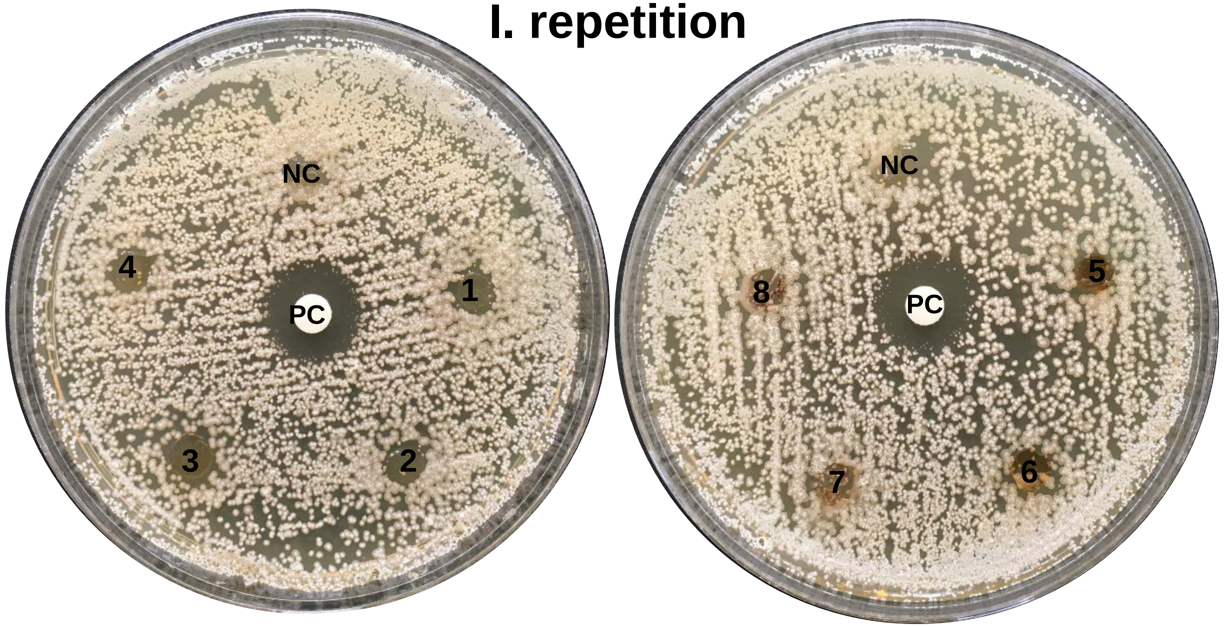

II. repetition

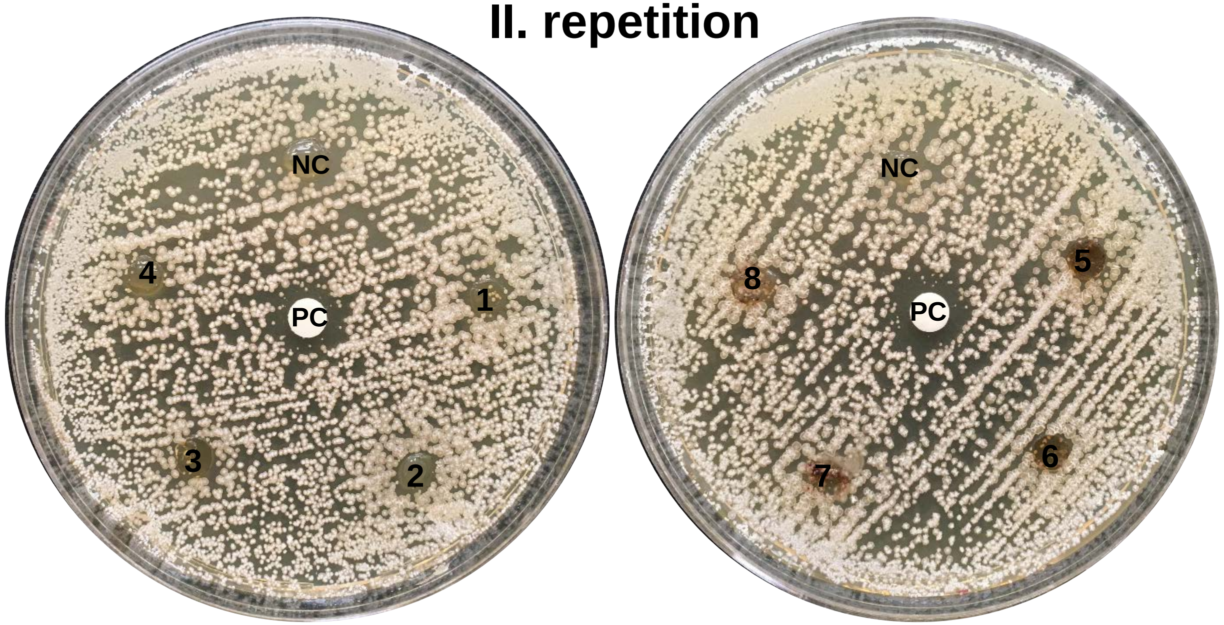

III. repetition

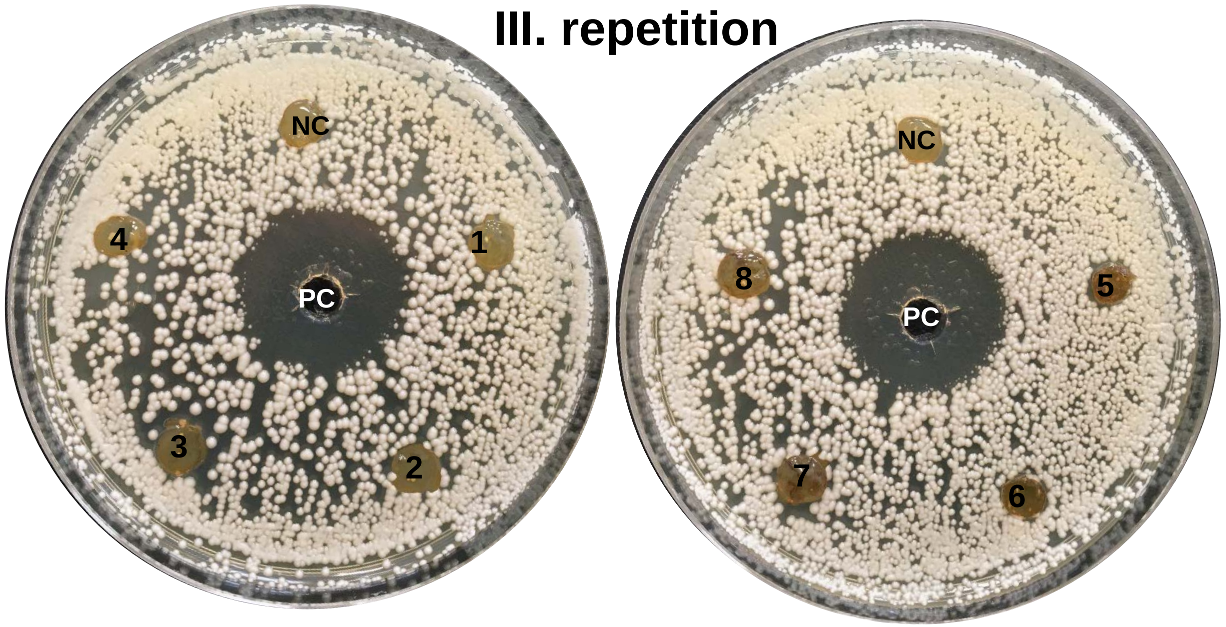

No	Tests	Zone diameter (mm)
**1**	**CS+EA (%1)**	Not available.
**2**	**CS+EA (%0.5)**	Not available.
3	CS+E (%1)	Not available.
4	CS+E (%0.5)	Not available.
**5**	**CS+EW(%1)**	Not available.
**6**	**CS+EW (%0.5)**	Not available.
7	Water (%1)	Not available.
8	Water (%0.5)	Not available.
NC	Negative control	Not available.
PC	Nystatin	23
